# Identification of myeloproliferative neoplasm drug agents via predictive simulation modeling: assessing responsiveness with micro-environment derived cytokines

**DOI:** 10.18632/oncotarget.8540

**Published:** 2016-04-01

**Authors:** Susumu S. Kobayashi, Shireen Vali, Ansu Kumar, Neeraj Singh, Taher Abbasi, Peter P. Sayeski

**Affiliations:** ^1^ Department of Medicine, Beth Israel Deaconess Medical Center/Harvard Medical School, Boston, MA, USA; ^2^ Cellworks Group, Inc., San Jose, CA, USA; ^3^ Cellworks Research India Pvt Ltd., Cellworks Group Inc., Bangalore, India; ^4^ Department of Physiology and Functional Genomics, University of Florida College of Medicine, Gainesville, FL, USA

**Keywords:** predictive modeling, simulated signaling, signal transduction, JaK kinase

## Abstract

Previous studies have shown that the bone marrow micro-environment supports the myeloproliferative neoplasms (MPN) phenotype including via the production of cytokines that can induce resistance to frontline MPN therapies. However, the mechanisms by which this occurs are poorly understood. Moreover, the ability to rapidly identify drug agents that can act as adjuvants to existing MPN frontline therapies is virtually non-existent. Here, using a novel predictive simulation approach, we sought to determine the effect of various drug agents on MPN cell lines, both with and without the micro-environment derived inflammatory cytokines. We first created individual simulation models for two representative MPN cell lines; HEL and SET-2, based on their genomic mutation and copy number variation (CNV) data. Running computational simulations on these virtual cell line models, we identified a synergistic effect of two drug agents on cell proliferation and viability; namely, the Jak2 kinase inhibitor, G6, and the Bcl-2 inhibitor, ABT737. IL-6 did not show any impact on the cells due to the predicted lack of IL-6 signaling within these cells. Interestingly, TNFα increased the sensitivity of the single drug agents and their use in combination while IFNγ decreased the sensitivity. In summary, this study predictively identified two drug agents that reduce MPN cell viability via independent mechanisms that was prospectively validated. Moreover, their efficacy is either potentiated or inhibited, by some of the micro-environment derived cytokines. Lastly, this study has validated the use of this simulation based technology to prospectively determine such responses.

## INTRODUCTION

The tumor micro-environment is a complex system of host cells that work, inadvertently, to promote the growth of neoplastic cells. Host cells within a typical tumor micro-environment include fibroblasts, endothelial cells, and immune cells [1, 2]. Within the bone marrow, the tumor micro-environment also includes osteoblasts, adipocytes, and various hematopoietic cells [3]. In the bone marrow, the micro-environment provides both the physical support, as well as a number of soluble factors, that serve to enhance neoplastic cell growth. The physical support is in the form of direct cell-cell contact and comes via the interaction of various extracellular proteins such as cadherins and growth factor receptors. The soluble, or paracrine factors, include various combinations of growth factors, cytokines, and/or chemokines, that similarly enhance neoplastic growth. Moreover, not only does the tumor micro-environment support neoplastic cell growth, but it also promotes resistance to eventual therapeutic drug agents [1, 4–6]. As such, therapeutic regimens may be most effective when the neoplastic cells themselves are directly targeted with one drug and the tumor micro-environment is targeted with another.

The Philadelphia chromosome negative myeloproliferative neoplasms (MPNs) encompass polycythemia vera (PV), essential thrombocythemia (ET), and myelofibrosis (MF). There are approximately 22 cases of PV, 24 cases of ET, and 1.46 cases of myelofibrosis for every 100,000 people, which amount to approximately 68,000 patients with PV, 74,000 with ET, and 4,500 with MF in the United States [7]. MPNs are characterized by similar pathological syndromes including excess production of blood cells from the bone marrow, pruritus, splenomegaly, and extramedullary hematopoiesis. Jak2 somatic mutations are found in about 95% of all PV patients and more than half of all ET and MF patients. Current frontline therapies for MPN include cyto-reductive agents such as hydroxyurea and the pan Jak1/2 small molecule inhibitor, Ruxolitinib [8]. Although these therapies provide significant palliative relief of some disease associated symptomologies, they do so for a relatively small percentage of treated patients. For example, in the case of Ruxolitinib, only 22% of treated patients (11 of 51) exhibited clinical improvement and not a single patient exhibited either partial or complete disease remission [9]. A very recent report has perhaps identified a mechanism for the poor clinical response that has been observed with Ruxolitinib. Specifically, when Jak2-V617F expressing cells were exposed to Ruxolitinib in culture, the drug was able to significantly reduce MPN cell viability [10]. However, when the same cells were co-cultured with bone marrow stroma derived from MPN patients, the ability of Ruxolitinib to reduce Jak2-V617F cell viability was lost [10]. Interestingly, this was not observed when the same Jak2-V617F MPN cells were co-cultured with bone marrow stroma taken from non-diseased, age matched, control subjects [10]. One reported difference between the MPN and non-diseased stroma was that the levels of inflammatory cytokines were significantly elevated in the MPN samples [10]. Thus, these results suggest that bone marrow stromal derived factors play a significant role in protecting Jak2-V617F positive cells from Ruxolitinib-induced cell death, thereby highlighting the role of the bone marrow micro-environment not only in the MPN disease pathogenesis, but also in resistance to subsequent Jak2 targeted therapies.

Predictive simulation modeling is an emerging technology in the realm of personalized medicine. A computational simulation avatar is created via genomic profiling information that is derived for a given cell line or patient biopsy. A digital library of molecularly targeted drugs is tested on this individual avatar (N=1), where drug agents are combined at different doses via simulation-based computation, resulting in analyses of the effects of a large number of drug combinations on various tumor phenotypes, including cell proliferation, apoptosis, and viability. A critical feature of this approach is that the drug library can be composed of drugs that have been approved for other indications and/or new investigative drug agents. The assessment of drug impact can be done under variations of the bone marrow micro-environment from the paracrine perspective, and along with the genomics driven autocrine loops, can provide insight into factors that are interfering with drug response. Shortlisted drugs and drug combinations are then experimentally validated on immortalized cell lines that represent the disease of interest or patient cells *ex vivo*. This predictive simulation approach differs from other biological modeling methodologies in that it incorporates integrated cancer physiology networks that cover all disease phenotypes in the simulation and provides semi-quantitative outputs and trends [11–13]. The network is manually aggregated from experimental data and addresses issues of contradictory datasets via evaluation of both the quality and context of the experimental data. In addition, the network is continuously updated with information from new reports using a regression based engineering methodology with stringent quality control. Thus, this semiconductor engineering based methodology creates a dynamic and transparent disease physiology model that makes feasible, a large quantum of simulation based drug combination hypotheses that can then be prospectively validated.

Given the importance of the tumor micro-environment on both MPN disease pathogenesis and drug resistance, we hypothesized herein that predictive simulation modeling could be employed in order to better understand the role of inflammatory cytokines on drug efficacy and resistance, and to develop a methodology to tease out the causal factors for enhancing or reducing the response of the prospectively validated shortlisted drug combinations. To test this, we selected two representative MPN cell lines, Human erythroleukemia 92.1.7 (HEL) and SET2, and obtained their genomic profile. The HEL cell is largely defined by the Jak2-V617F mutation, along with deletion of CDKN2A, RB1 and TP53 [14–16]; while the SET-2 cells are defined by Jak2 mutations as well as TP53, and additional mutations and copy number variations as reported in C-BioPortal [16]. Simulation avatars were then created for each cell line and the signaling networks were simulated, both with and without inflammatory cytokines tumor necrosis factor alpha (TNFα), interferon gamma (IFNγ) and interleukin 6 (IL-6). We identified two drugs (the Jak2 kinase inhibitor, G6, and the Bcl-2 inhibitor, ABT737) that could significantly reduce MPN cell viability when used alone and their effects were synergistic when used in combination. Furthermore, we found that the inflammatory cytokine TNFα increased the sensitivity of the single drug agents and their use in combination. On the other hand, IFNγ reduced sensitivity of the individual drugs while having no impact on the combination, while IL-6 was found to not impact the drug sensitivity in these profiles.

## RESULTS

### Predictive simulation modeling

**Figure 1:**
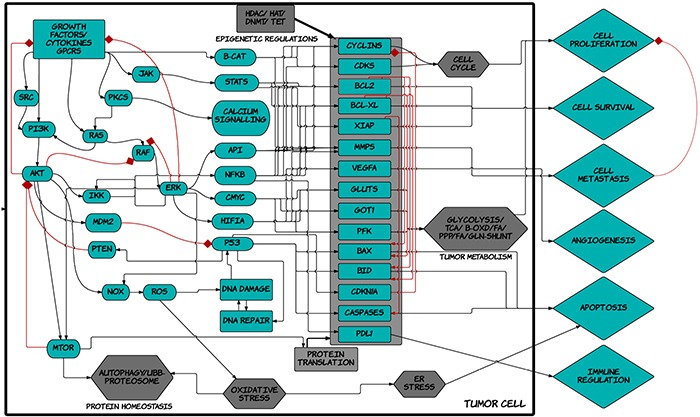
Schematic diagram of ™CELLWORKS cancer cell platform A high level schematic diagram of the key pathways including inputs such as growth factors, cytokines, kinases, adaptors, transcription and translation machineries; cellular processes such as protein homeostasis, oxidative and ER stress, DNA repair, epigenetic regulations, tumor metabolism, cell cycle regulation; and the various tumor phenotypes of proliferation, viability, metastasis, angiogenesis, immune regulation, apoptosis; present in the virtual tumor cell technology. The Cellworks Oncology Platform was customized to create the virtual MPN cell lines HEL and SET2.

The simulation model used herein includes representation of important signaling pathways implicated in cancer as detailed in the methods below (Figure 1). The SET2 and HEL cell line models that are representative of Jak2-V617F driven MPN were created as described in the methods. The mutation and copy number variation (CNV) aberration data for these cell lines were obtained from the literature and cancer portals such as Sanger and cBioportal [14–16, Appendix 1]. SET2 cells, besides having the Jak2-V617F mutation, also has mutations in RPTOR, TP53, CCNA2, NOTCH2, EGF, MAP2K1 as well as multiple gene amplifications and deletions as listed in Appendix 1. Definition for the HEL cell line obtained from Sanger comprised of mutations in Jak2, TP53, CDKN2A, as well as RB1 deletion and E2F1 amplification. There are also many CNVs (Appendix 1). Once defined, the cell line simulation models were validated at the biomarker and phenotype level against available experimental results on these cell lines.

### Retrospective validation of virtual HEL and SET2 cell lines

**Figure 2:**
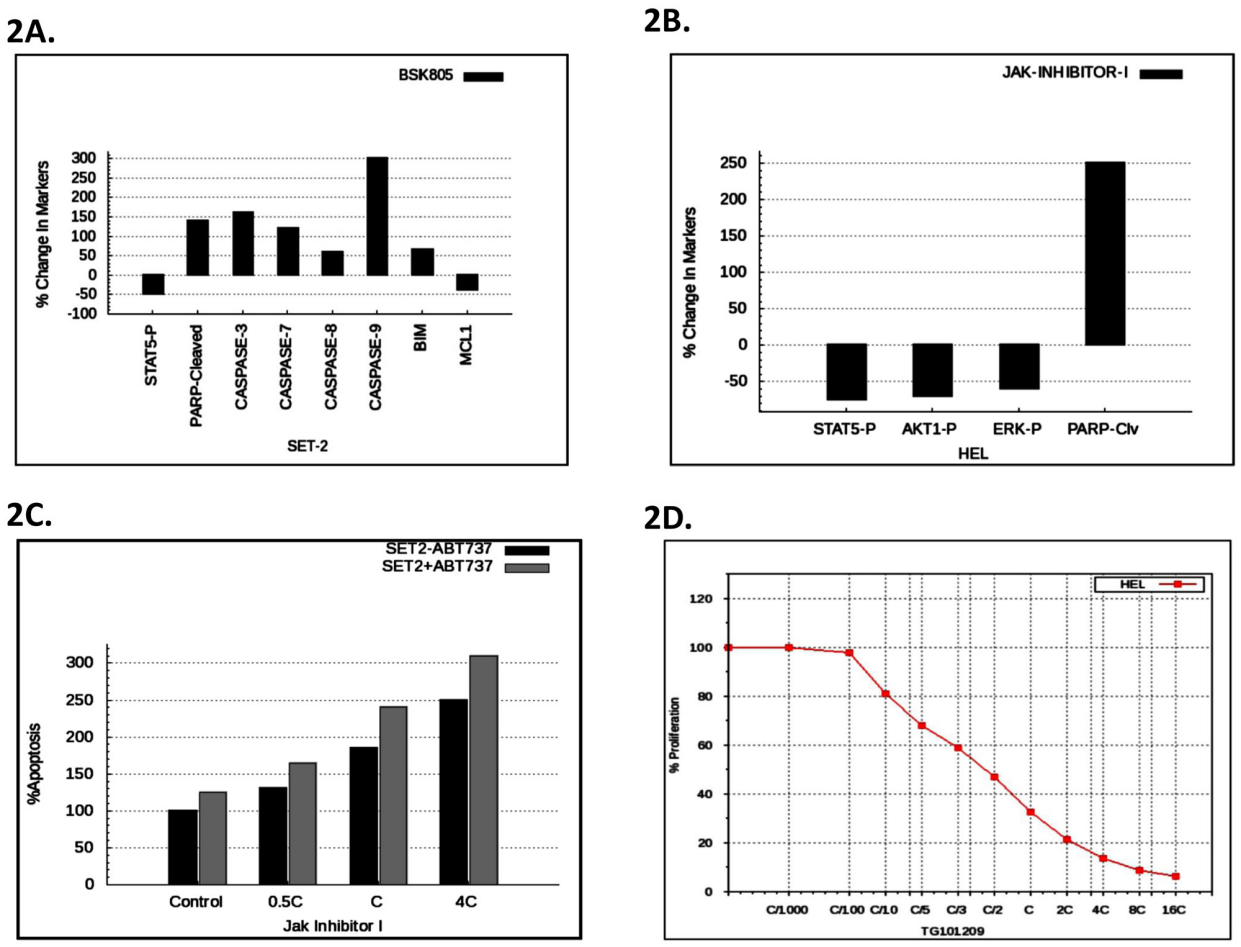
Predictive vs. experimental correlation of various JAK inhibitors on HEL and SET2 cells **A.** The predictive plot with % change in protein levels for the indicated disease markers expressed in SET2 cells after virtual treatment with BSK805. **B.** The simulation avatar predicted effect of JAK inhibitor-1 on pSTAT, pAKT, pERK, and cleaved PARP in HEL cells. **C.** The simulation avatar-predicted dose response effect of JAK inhibitor-1, with or without ABT737, on apoptosis in SET cells. ‘C’ is the concentration of the drug at 60% on-target inhibition. **D.** Simulation avatar predictive dose response curve for the JAK inhibitor, TG101209, on HEL cell viability with varying the C concentration (60% on-target inhibition) from C/1000 to 10C.

In order to determine the confidence of the virtual MPN HEL and SET2 cell lines, we evaluated the correlation of predicted outcomes with that various known experimental datasets in the literature including Rubert *et al.* [17], Will *et al*. [16], and Pardanani *et al.* [18]. Figures 2A shows the predictive data for the effect of NVP-BSK805 (a Jak2 inhibitor) on the Jak2-V617F dominant SET2 cell line. In particular, the simulation modeling predicted increases of the apoptotic markers cleaved PARP, Caspases-3, -7 -,8 and -9, BIM as well as decreases in phosphorylated STAT5 and anti-apoptotic MCL1 (Figure 2A). These predictions aligned very well with the previously published experimental data for this cell line and this drug effect [17].

Figure 2B depicts the predictive simulation data of the effect of JAK Inhibitor-1 treated HEL cells. Specifically, the simulations predicted reductions of phosphorylated STAT5, AKT, and ERK, as well as increased cleaved PARP. We found that the predictive data matched with the retrospective experimental results of JAK Inhibitor-1 treated HEL cells [16]. Will *et al*. also presented results on the enhancement of efficacy of JAK inhibitor-1 on apoptosis in SET2 cells, by the inclusion of the BCL2 inhibitor, ABT737 [16]. This combination of JAK inhibitor-1, along with BCL2 inhibitor ABT737, was therefore tested predictively on the virtual SET2 cell line. The predictive results are presented as Figure 2C. We found that the predictive results matched the experimental results [16], thereby validating the virtual SET2 cell line, retrospectively.

Pardanani *et al.* similarly tested the ability of the Jak2 kinase inhibitor, TG101209, to inhibit HEL cell proliferation [18]. Our predictive simulation modeling (Figure 2D) correlated very well with the previously generated experimental data [18], thereby validating the virtual HEL cell line in this regard.

Thus, when taken together, the data in Figure 2 validated, on a retrospective basis, the virtual SET and HEL cell lines. Consequently, the avatars for these two cell lines can be digitally leveraged, in order to better understand MPN biology.

### Prospective validation of predictive simulations and synergistic interaction between ABT737 and G6

**Figure 3:**
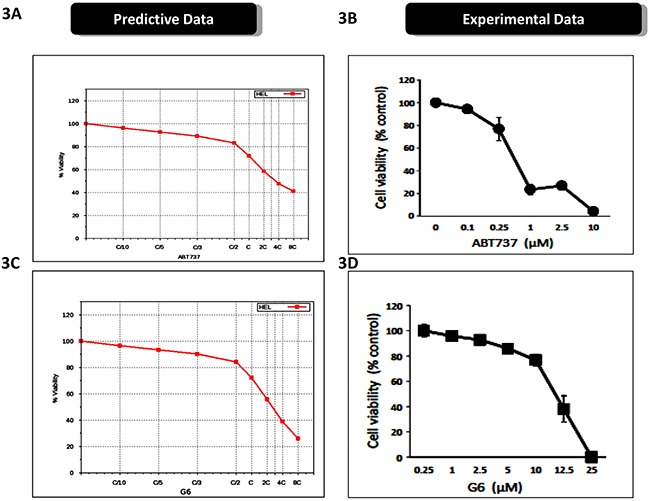
Prospective validation of predictive simulations and synergistic interaction between ABT737 and G6 **A.** The virtual HEL cell line was simulated with increasing concentrations of ABT737 (C/10 to 8C) and the percent of viable cells was plotted as a function of drug concentration. ‘C’ is the predicted concentration of the drug at 60% on-target inhibition. **B.** Prospective validation of the drug effect whereby HEL cells were treated with the indicated concentrations of ABT737 and viability was measured three days later. Each data point was measured in triplicate. **C.** The virtual HEL cell line was simulated with increasing concentrations of the JAK inhibitor (C/10 to 8C), G6, and the percent of viable cells was plotted as a function of drug concentration. ‘C’ is the predicted concentration of the drug at 60% on-target inhibition. **D.** Prospective correlation of the G6 effect whereby HEL cells were treated with the indicated concentrations of G6 and viability was measured three days later. Each data point was measured in triplicate. Shown is one of three (B) or two (D) representative results.

The predictive simulation modeling and the subsequent therapeutic drug simulations indicated that the Bcl2 inhibitor, ABT737, and the pre-clinical Jak2 kinase inhibitor, G6, would be efficacious against Jak2 driven pathogenesis, when used alone and synergistically when used in combination. ABT737 is a BH3 mimetic and inhibits several proliferative genes including Bcl-2, Bcl-xL, and Bcl-w [19]. G6 is a preclinical Jak kinase inhibitor with a Jak selectivity profile of Jak2>Jak3>>Jak1>>>Tyk2 [20]. The STAT selectivity profile for this compound is STAT5>STAT3>STAT1, and its principal mechanism of action is induction of apoptosis [21]. Figure 3A shows the ABT737 predictive simulation results indicting a dose-dependent decrease on HEL cell viability, while Figure 3B shows the validation of these results on cultured cells. Figure 3C shows the predictive simulation results of G6 on HEL cell viability while Figure 3D displays the prospective validation of those forecasts.

**Figure 4:**
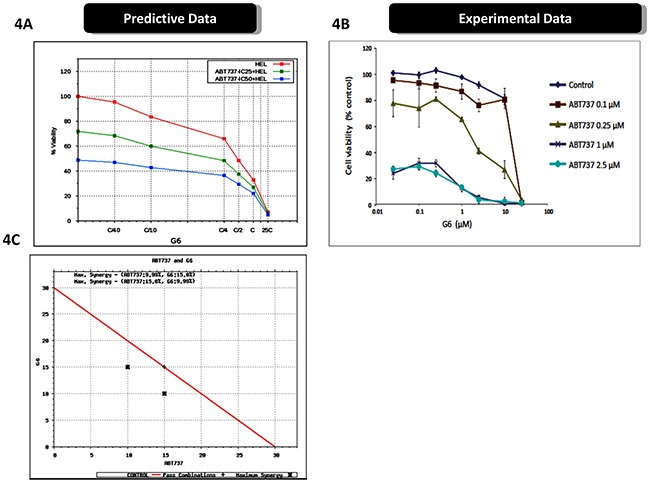
Prospective validation of predictive simulations avatars and synergistic interaction between ABT737 and G6 **A.** The virtual HEL cell line was simulated with the indicated concentrations of ABT737 (fixed dose of drug IC25 and IC_50_ viability) and dose response of varying dose of G6 with ‘C’ being the predicted concentration of the drug at 60% on-target inhibition, and cell viability was then plotted as a function of drug concentrations. **B.** Prospective validation of the drug effects on cultured HEL cells treated with the indicated concentrations of ABT737 and G6. Cell viability was then measured three days later. Each data point was measured in triplicate. Shown is one of three representative results. **C.** An isobologram showing the IC_50_ concentrations of the two drugs (red line), plotted as a function of individual drug concentrations. The black crosses indicate the sub-IC_50_ concentrations that were required to reduce the cell viability by one-half, thereby indicating synergy between ABT737 and G6.

Figure 4A shows the predictive effect of the drugs, when used in combination, on HEL cell viability. The simulations predicted a synergistic relationship between the two compounds. To validate these data, we treated HEL cells with various combinations of ABT737 and G6, and subsequently measured HEL cell viability. We found that the experimental results closely paralleled the virtual simulations and thus validated the predicted results (Figure 4B). In order to determine if the relationship between ABT737 and G6 was in fact synergistic, we simulated various dosages of G6 and ABT737 in combination, in order to identify the minimum concentration that could achieve at least 50% reduction in cell viability. The results from these simulation studies are plotted in the isobologram shown in Figure 4C. The isobologram plot has the concentration of the Jak2 inhibitor, G6, on the y-axis and the concentration of the BCL2 inhibitor on the x-axis. The red line connects the IC_50_ concentrations of the two drugs. Combinations of the drugs at the varied doses indicates that a combination of Jak2 inhibitor, along with BCL2 inhibitor at half the IC_50_ G6 dose, along with 1/3rd of the ABT737 dose, indicated by the black cross on the left, could achieve the same 50% inhibition as the individual IC_50_ doses. Similarly 1/3rd of G6 dose with half of the ABT737 dose, as indicated by the black cross on the right, also gave an IC_50_ inhibition, indicating that these two drugs are synergistic in reducing HEL cell viability.

Collectively, predictive cellular modeling and subsequent drug simulations indicated that ABT737 and G6 would significantly decrease HEL cell viability when used individually, and synergistically, when used in combination. The subsequent prospective validation on cultured HEL cells validated those predictions. As such, these studies validate the virtual HEL cell line and how two investigative drug agents affect it.

### Inflammatory cytokines modulate the effects of ABT737 and G6 on cell viability

**Figure 5:**
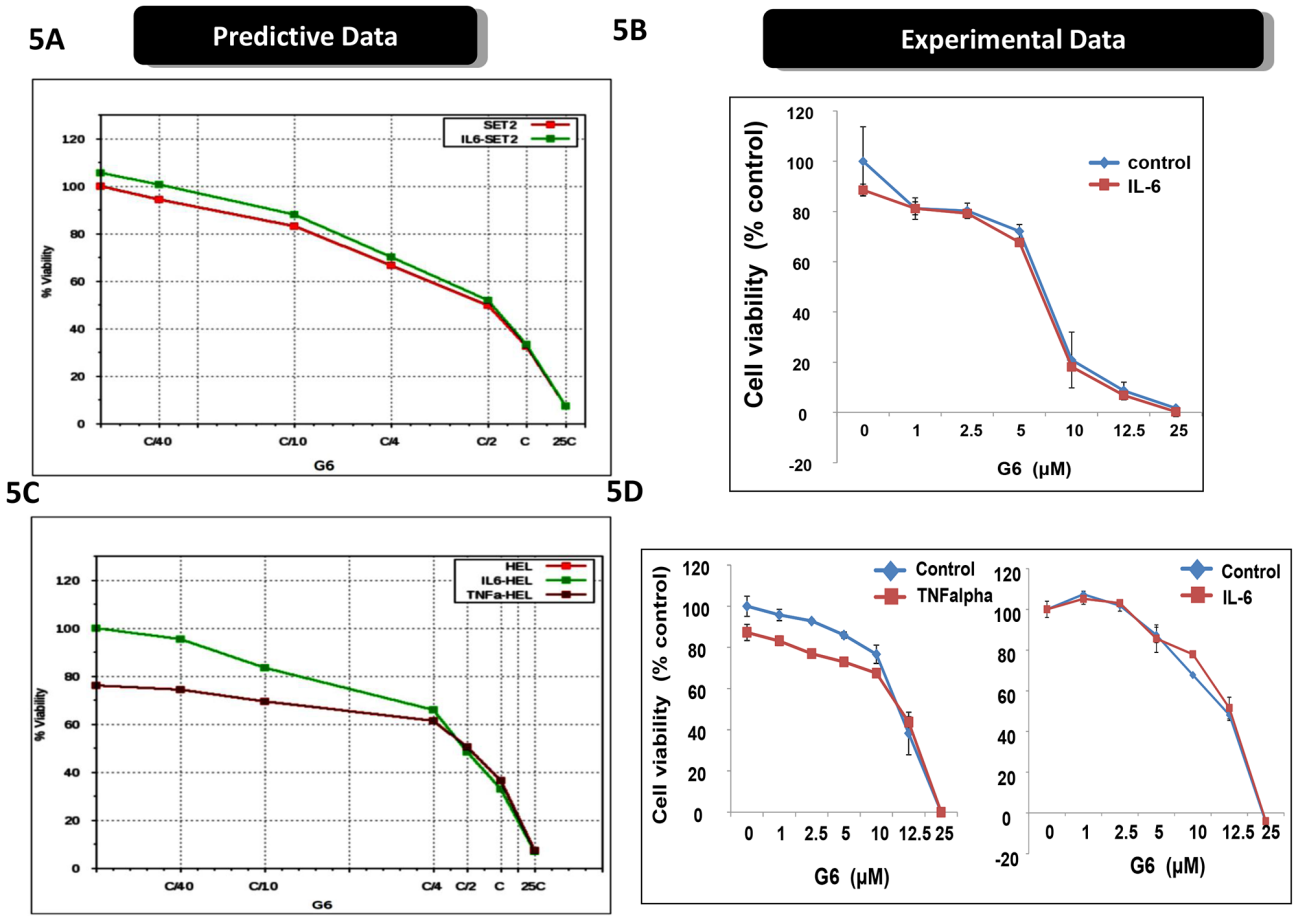
Inflammatory cytokines differentially affect the efficacy of G6 **A.** The virtual SET2 cell line was simulated with the indicated concentrations G6, both in the presence and absence of IL-6. ‘C’ is the predicted concentration of the drug at 60% on-target inhibition. The percent of viable cells was then plotted as a function of G6 concentration. **B.** Prospective validation of the IL-6 effect on cultured SET2 cells whereby the percent of viable cells was plotted as a function of G6 concentration, both in the presence and absence of IL-6. Each data point was measured in triplicate. Shown is one of three representative results. **C.** The virtual HEL cell line was simulated with the indicated concentrations of G6, both in the presence and absence of either IL-6 or TNFα. The percent of viable cells was then plotted as a function of G6 concentration. **D.** Prospective validation of the cytokine effect on cultured HEL cells whereby the percent of viable cells was plotted as a function of G6 concentration, both with and without cytokine. Each data point was measured in triplicate. Shown is one of three representative results.

**Figure 6:**
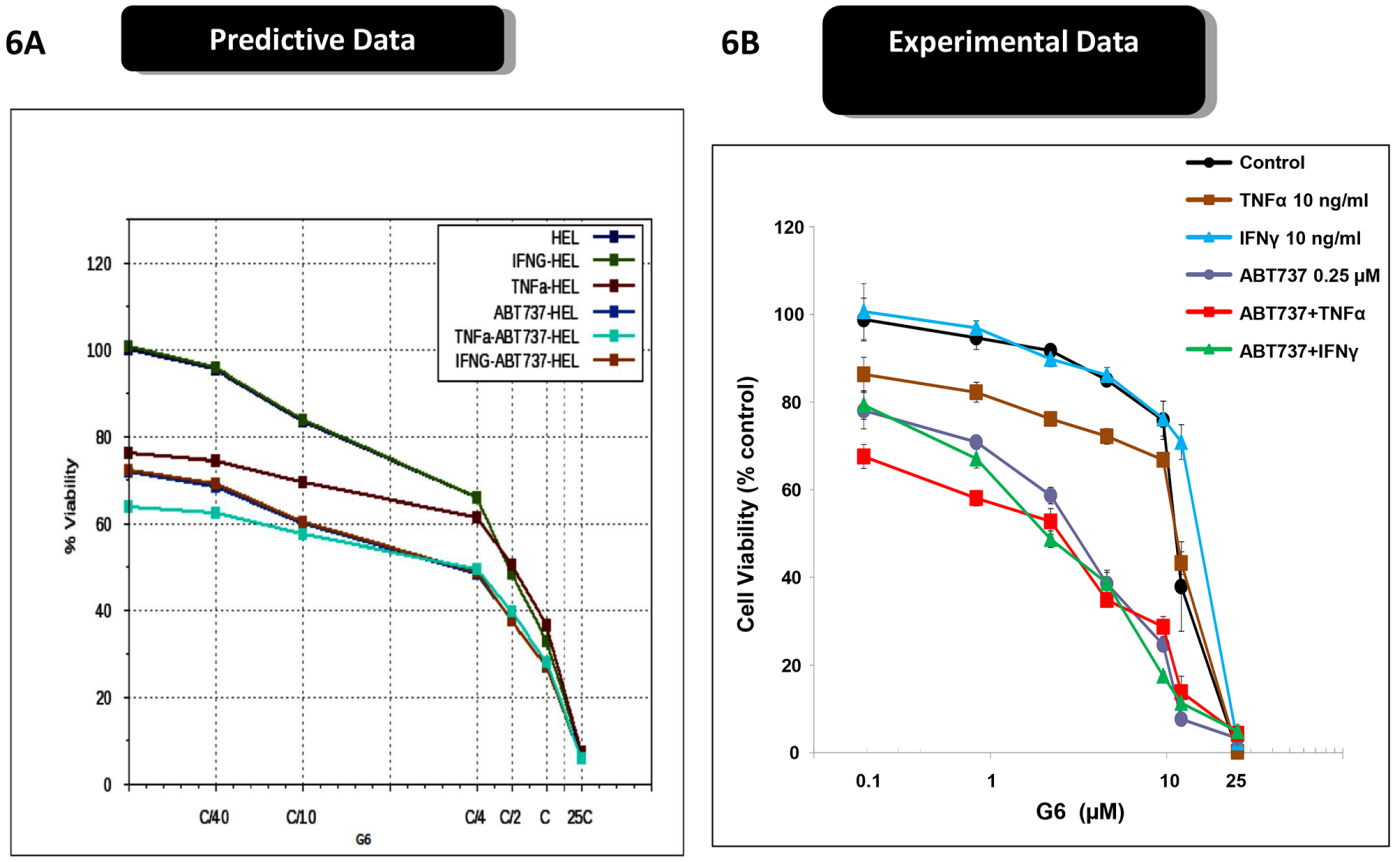
Inflammatory cytokines differentially affect the efficacy of ABT737 and G6 **A.** The virtual HEL cell line was simulated with the indicated concentrations G6 and ABT737, both in the presence and absence of either TNFα or INF-γ. The percent of viable cells for each condition was then plotted as a function of G6 concentration. **B.** Prospective validation of the cytokine effect on cultured HEL cells whereby the number of viable cells was plotted as a function of G6 concentration. Each data point was measured in triplicate. Shown is one of three representative results.

Cytokine storm is a common syndrome within MPN patients. It is characterized by increased levels of inflammatory cytokines, both in the bone marrow and the peripheral blood [22–25]. Within the bone marrow, inflammatory cytokines such as IL-6 and TNFα contribute to a number of deleterious actions including the development of drug resistance. For example, previous reports have shown that inflammatory cytokines, *per se*, can render MPN cells resistant to Jak kinase inhibitors including Ruxolitinib and atiprimod [10, 26]. Interestingly, because of the distinct mechanisms of actions for ABT737 and G6, our predictive simulation modeling indicated these investigative drugs would not be adversely affected by inflammatory cytokines. To explore this, we first examined receptor expression for IL-6, TNFα, and IFNγ, on both SET-2 and HEL cells. We found that both cell lines lacked IL-6 receptor expression so therefore, this cytokine would serve as an appropriate negative control. However, both cell lines were found to express the appropriate compliment of receptors for both TNFα and IFNγ. Moreover, our predictive simulations indicated that these two inflammatory cytokines would not hinder the actions of ABT737 and G6. Figure 5A shows the predicted effect of G6 on SET-2 cell viability, both in the presence and absence of IL-6. Given the lack of IL-6 receptor expression on SET-2 cells, our simulations indicated that IL-6 would not impact the ability of G6 to reduce SET-2 cell viability. This was confirmed experimentally when SET-2 cells were treated with increasing concentrations of G6, both in the presence and absence of IL-6 (Figure 5B). With respect to HEL cells, our predictive modeling indicated that IL-6 would similarly be without effect while TNFα would potentiate the effect of G6 on reducing HEL cell viability (Figure 5C). This was confirmed experimentally whereby the IL-6 was found to be without effect whereas TNFα increased the sensitivity of the cells to concentrations of G6 that were between 1 - 10 μM (Figure 5D). When ABT737 was modeled into the simulations, we found that TNFα potentiated the effect of not only G6, but also the combination of G6 and ABT737 (Figure 6A). When this was tested in cultured cells, we observed a similar result (Figure 6B). IFNγ on the other hand decreased the sensitivity of G6, post a threshold concentration when used alone (Figure 6B), and did not show much impact on the efficacy when used in combination.

Overall, the results in Figures 5 and 6 indicate that, rather than diminishing the inhibitory potential of G6 and ABT737, TNFα in fact augments them while IFNγ and IL6 did not show much impact. Thus, the combination therapy of G6 and ABT737 is not only highly effective at reducing HEL cell viability, but also withstands cytokine induced drug resistance normally caused by TNFα.

## DISCUSSION

Dysregulated Jak2 kinase signaling has been implicated in a number of human pathologies including hypertension, diabetes, and cancer [27]. Within the bone marrow, dysregulated Jak2 signaling is known to be a causative agent in MPN [27, 28]. The constitutive Jak2-dependent signaling drives excessive proliferation and differentiation of cells within the myeloid lineage. With respect to potential drug therapies, unfortunately, over the past few years, six Jak2 small molecule inhibitors have been abandoned in clinical studies for either a lack of efficacy and/or severe adverse events [29, 30]. These include drugs developed by Lilly (Gandotinib), Bristol-Myers Squibb (BMS-911543), Sanofi Aventis (Fedratinib), Cephalon (Lestaurtinib), AstraZeneca (AZD1480) and Exelixis (XL019). Moreover, for the only FDA approved Jak2 inhibitor, Ruxolitinib, studies have clearly demonstrated an acquired drug resistance that involves inflammatory cytokines [10, 26]. Thus, identifying therapeutic agents that can reduce MPN cell viability, especially in the face of inflammatory cytokines present in the bone marrow microenvironment, is highly desirable. Here, we used predictive simulation modeling in order to identify drug agents that could not only reduce MPN cell viability, but do so despite bone marrow derived inflammatory cytokines. To this end, we found that the Jak2 kinase inhibitor, G6, and the Bcl-2 inhibitor, ABT737, significantly reduced MPN cell viability when used alone, and their effects were synergistic when used in combination. Furthermore, we found that the inflammatory cytokine, TNF-α, increased the sensitivity of the single drug agents and their use in combination. As such, these results have defined a novel set of drug agents that have a differentiating element when compared to currently available front line therapies; namely, an ability to be more, rather than less potent, in the face of an inflammatory cytokine in the form of TNF-α.

**Figure 7:**
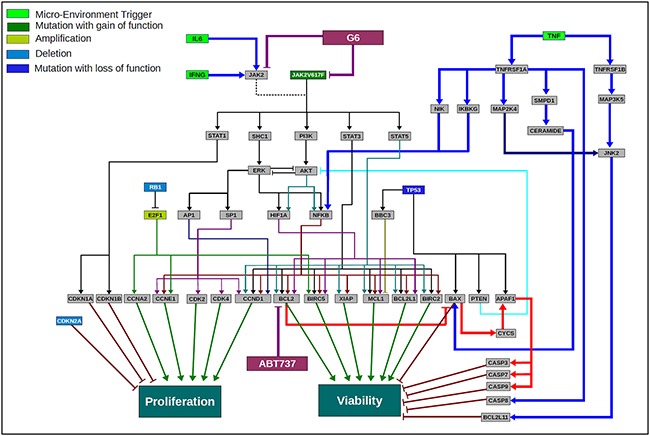
Scientific rationale schematic for the HEL disease network and the impact of the cytokines in the tumor microenvironment on the efficacy of G6 and ABT737 combination therapy A network schematic depicting the key pathways and aberrations present in HEL cell line that has JAK2-V617F as a key driver mutation along with a mutation of P53, deletion of RB1 and CDKN2A and amplification of E2F1. A constitutively activated JAK2 mutant activates various downstream pathway loops leading to activation of ERK, AKT and key transcription factors including STAT3, STAT5, NFkB, AP1 and others. This along with the other aberrations converge at a highly proliferative phenotype and increased cell viability. The cytokine modulated pathways are depicted in terms of how they interact with the JAK2 driven disease network. IFNγ and IL6 converge via JAK2 wild type signaling and therefore are not showing a significant impact in changing the outcome of G6 efficacy since JAK2 is mutated and constitutively activated. TNFα on the other hand activates NFkB, but also activates the pro-apoptotic pathway loop, thereby enhancing the efficacy of the JAK2 inhibitor and the combination. The inhibitors G6 and ABT737 are also shown inhibiting their respective targets and the schematic highlights the rationale for the use of these two drugs in combination in a JAK2-V617F driven MPN disease network.

In PV, >90% of the patients harbor the Jak2-V617F mutation and in most instances, this is both the key and only aberration. In such profiles, there is a Jak2 driven up-regulation of anti-apoptotic genes BCL2, MCL1, BCL2L1 [18, 28]. There is also an increased activation of proliferative genes such as CCND1, CCNE1 and CCNA1 and cell cycle kinases including CDK2/4 [31]. Figure 7 depicts the signaling networks that are dysregulated in a model of Jak2-mediated pathogenesis and the impact of IL-6, IFN-γ, and TNF-α on those signaling networks. In addition, the schematic shows the nodes of inhibition for the BCL2 inhibitor ABT737 and Jak2 kinase inhibitor G6. In a Jak2-V617F driven profile, the Jak2 downstream signaling via STAT3, STAT5, PI3K/AKT, SHC1/ERK and STAT1 are activated [30, 31]. In this schematic of HEL cell signaling, besides the JAK2 mutation, there is also a loss of function mutation of TP53, deletion of RB1 and CDKN2A as well as amplification of E2F1, all leading to increased proliferation and viability. G6 inhibits the dominant Jak2 signaling in this Jak2 mutant driven network. BCL2, a key anti-apoptotic protein is activated downstream of the Jak2-V617 mutation via STAT3, STAT5, and NF-kB. Thus, inhibiting this downstream signal, along with a more direct inhibition of the driver pathway, synergize to inhibit cell proliferation and viability as we prospectively validated in MPN representative cell lines. One advantage of the G6 and ABT-737 combination therapy is that it synergizes by inhibiting both proliferation and viability; specifically, G6 inhibits the proliferative impact of the Jak2 mutation itself while ABT-737 targets the anti-apoptotic pathways. Moreover, the Jak2-V617F mutation activates multiple anti-apoptotic proteins, including MCL1. Inhibition of Jak2 by G6 inhibits MCL1 via downstream inhibition of its transcription factors [21] and therefore contributes further to enhancing induction of apoptosis and reducing tumor cell viability. This also addresses the possible resistance mechanism that can arise when MCL1 expression increases subsequent to BCL2 inhibition [32, 33].

Across the various cytokines measured in the plasma of MPN patients, eight chief cytokines have been described; MIP-1β, TNF-α, IL-6, GM-CSF, IFN-γ, G-CSF, IFN-α, and Rantes [24, 25, 34]. The observed concentration of these cytokines in MPN patients is higher than that in normal donors, consistent with other published reports [23]. Of these cytokines, we determined the impact of IL-6, TNF-α, and IFN-γ on our virtual cell lines, with subsequent experimental validation. Given the lack of IL-6 receptor expression on HEL and SET-2 cells, IL-6 was predictably without effect. The predicted and validated effect of IFNγ on drug efficacy was minimal. Lastly, we found that TNFα potentiated the efficacy of the drug agents when used either alone or in combination. As such, these studies define novel interactions between inflammatory cytokines and experimental drug efficacy, which can potentially be exploited for circumventing mechanisms of cytokine-induced drug resistance.

The predictive simulation modeling that was employed in this work integrates signal transduction, epigenetic regulation, protein homeostasis, metabolic pathways, and other phenotypes representing not just MPN, but all cancers (Figure 1). The platform as demonstrated in this study, has the ability to predict cellular outcomes and the subsequent identification of personalized therapeutic assets for any given profile, whether it is a cancer cell line such as those used herein or a human patient sample [11–13]. The workflow utilizes drug agents that can either be FDA approved (i.e., ABT737) or under investigation (i.e., G6). As such, FDA approved drugs can readily be repurposed for new indications which provides a rapid path to clinical translation, while investigational drugs can rapidly be screened *in silico* in various profiles and disease models across a large number of phenotypes and biomarkers. This can provide an in-depth insight into efficacy and mechanistic details without putting in extensive resource, time, and efforts into running experiments. The data provided herein have been used as the foundation for a personalized medicine approach for several hematological disorders. Specifically, peripheral blood and/or bone marrow samples are being collected from myelofibrosis, multiple myeloma, acute myeloid leukemia, acute lymphoblastic leukemia, and myelodysplastic syndrome patients under an IRB approved protocol (NCT02435550). After collection, the samples are being subjected to next-generation sequence (NGS) analysis, protein mutation analysis, and metabolomic analysis. The profiling data are then being fed into the predictive simulation modeling, where a short list of novel drug combinations is subsequently generated for each patient. The drug combinations are then being validated on *ex vivo* cultures using the same patient's primary cells. The most efficacious therapy is then being tested in the patient. Consequently, the studies described in this current work have served as the basis for a hematological-based, precision medicine campaign that can readily be leveraged into other cancers as well.

In summary, this work identified two investigative drug agents that act significantly when used alone and synergistically when used in combination, to reduce the viability of two MPN model cell lines. We found that their efficacy is potentiated by some inflammatory cytokines that are typically elevated in MPN. Thus, by accurately predicting responses of cells to targeted agents *a priori* in context of their genomics and microenvironment, the *in silico* simulation model provides an innovative approach to precision medicine for not only MPN, but other neoplastic disorders as well.

## MATERIALS AND METHODS

### Cancer simulation model

The simulation experiments and analyses were performed using the predictive tumor model, a comprehensive and dynamic network of signaling, and metabolic pathways in the context of cancer physiology as described [35]. The simulation model used herein, as depicted in a very high level schematic of the network in Figure 1, includes growth factors like EGFR, PDGFR, FGFR, c-MET, VEGFR and IGF-1R; cytokine and chemokines like IL1, IL4, IL6, IL12, TNFα, IFNγ; GPCR mediated signaling pathways; mTOR signaling; cell cycle regulations; tumor metabolism; oxidative and ER stress; representation of autophagy and proteosomal degradation; DNA damage repair, p53 signaling and apoptotic cascade. The network also includes epigenetic regulation mechanisms and shows all the phenotypes that are modeled and can be assessed. The referenced current version of cancer model includes more than 4700 intracellular biological entities and ~6500 reactions representing their interactions regulated by ~25000 kinetic parameters. This comprises a comprehensive and extensive coverage of the kinome, transcriptome, proteome, and the metabolome. There are 142 kinases and 102 transcription factors modeled in the system.

### Simulation protocol

The simulation protocol for the testing of the drug combinations in the virtual MPN cell line models for HEL and SET2 in context of the different microenvironment based cytokine variation consisted of the following steps:

1. Control stage: The model is simulated for 50,000 seconds simulation time during which the different species (biological entities including enzymes, receptors, metabolites etc.) attain a steady state concentration. This concentration is dependent upon the balance between the rate of reaction nodes producing the species and the reaction nodes utilizing/degrading the species. This is the un-triggered control baseline.
2. Disease stage: At simulation time of 50,000 seconds, the genomic aberrations for a profile (cell line or patient biopsy) is overlaid on the control network through representing the aberrations as increased or decreased expression/activity of genes or proteins to represent copy number variations and mutations respectively. The system is then simulated further to 125,000 seconds simulation time. During this time, the system attains a new steady state which aligns to the network dynamics of the tumor cell line or patient profile depending on the genomic input file.
3. Disease + Drug Stage: Once the disease baseline is created, the drug is introduced in the system at 125,000 seconds by perturbing the target reaction nodes to represent the drug mechanism of action. The system is simulated further until 200,000 seconds simulation time. The percentage change seen in values of the biomarkers and phenotypes such as viability and proliferation index from the disease baseline give the read out for the therapeutic potential of the drug.
4. Disease + Microenvironment + Drug: To the disease baseline we first add in the changed cytokine and chemokine paracrine inputs, either cumulatively or individually, and simulate to create the disease baseline variant with the microenvironment. The drug can then be simulated on this modified disease baseline in presence of the microenvironment factors to assess response, and compare against the response without the microenvironment factors.

### Creation of cell line models

To create the MPN cell line models SET2 and HEL, we used the simulation protocol described above. Genetic profiles (mutation and CNV) of these cell lines were derived from sequencing and cytogenetic data in the literature. Data obtained from the cBioportal and Sanger databases were used as input files to trigger the system to a neoplastic disease state (Appendix 1 has the profile definitions). Oncogenic mutations are modeled as gain of function at the protein activity level and mutations in tumor suppressors are modeled as loss of function. Deletion and amplification aberrations are modeled by over-expressing or knocking down the expression of the gene at the level of its transcription. These representations of the genomic aberrations are input in the form of a bio-assembly code and overlaid on the control network that is then simulated to achieve the dynamic disease state.

Disease phenotypes such as proliferation and viability indexes can be assessed to ascertain disease induction and severity. Disease phenotype indices are defined in the tumor model as functions of biomarkers that are involved in regulating these aspects of the tumor cell. The proliferation index is an average function of the active CDK-Cyclin complexes that define cell cycle check points and are key for regulating overall tumor proliferation potential. The biomarkers include: CDK4-CCND1, CDK2-CCNE, CDK2-CCNA and CDK1-CCNB1. The biomarkers have been given a certain weightage and permutations of the same have been tested to reach to an index definition that gives the maximum correlation with the experimentally reported trend for proliferation across a large number of studies.

The viability index is a ratio of two sub-indices: Survival over Apoptosis. The components for each of the sub-indices have been selected based on their regulation and convergence towards these endpoints. The biomarkers constituting the survival index include: AKT1, BCL2, MCL1, BIRC5, BIRC2 and XIAP. All these biomarkers have been well reported to supporting tumor survival. The apoptosis index comprises of: BAX, CASP3, NOXA and CASP8. The overall cell viability index is then calculated as a ratio of survival index/apoptosis index. The weightage of each biomarker is decided to achieve a maximum correlation with experimental trends for the end points.

### Simulation of drug effect

To simulate the effect of a drug in the *in silico* tumor model, the targets and mechanism of action of the drug are determined from published literature and defined through a specialized syntax. The drug concentration is explicitly assumed to be post-ADME (Absorption, Distribution, Metabolism and Excretion), at the site of action and the dose response of the drug entails varying the percentage target inhibition of the primary drug target that could equate to different concentrations of the drug in the *in vivo* setting.

### Experimental methods

#### Cell culture

HEL cells were purchased from ATCC (Rockville, MD). SET-2 cells were obtained from DSMZ (Braunschweig, Germany). The cells were cultured in RPMI-1640 medium containing 10% fetal bovine serum at 37°C in a 5% CO_2_ humidified atmosphere and passaged at least two times per week. The Jak2 inhibitor, G6, was obtained from the NIH/NCI drug repository (NSC33994). The Bcl2 inhibitor, ABT737, was obtained from Selleck Chemicals. The IL-6, TNFα, and IFNγ were purchased from R&D Systems (Minneapolis, MN).

#### Measurements of cell viability

Log phase cells were seeded in 96-well plates at 30,000 cells per well in 100 μl of media containing either 0.25% DMSO or varying concentrations of drug. Cell viability was determined 68-72 hours later via the CellTiter 96AqueousOne Solution Reagent from Promega (Madison, WI) according to the manufacturer's protocol. Specifically, 20 μl of reagent was added to each well and plates were incubated for 2-3 hours at 37°C and absorbance was then read at 490nm using a 96-well plate reader. Data are expressed as a percentage of vehicle-treated cells (0.25% DMSO), which was arbitrarily defined as 100% viable.

#### Statistics

All results were expressed as mean ± SD. Statistical comparisons were done using the Student's *t*-test as well as a repeated measures analysis of variance followed by Bonferroni and Student-Newman-Keuls post hoc tests for multiple comparisons. P values of <0.05 were considered statistically significant. There is no statistical variation seen in the predictive results since it is based on a kinetic model that will achieve the same results with repeated perturbations. The variation can be seen by varying the perturbations/inputs.

## SUPPLEMENTARY TABLES


