# Supplementary material for: Identification of myeloproliferative neoplasm drug agents via predictive simulation modeling: assessing responsiveness with micro-environment derived cytokines
Source: Oncotarget. 2016 Apr 1;7(24):35989–6001. doi: 10.18632/oncotarget.8540 (PMC5094977; doi:10.18632/oncotarget.8540)
Supplement: Supplementary file 1 [file oncotarget-07-35989-s001.docx]

**SET-2 Cell Definitions Kobayashi *et al.***

|  | | |  |  |
| --- | --- | --- | --- | --- |
| **Sanger-Mutation** | | | | |
|  |  |  |  |  |
| **Gene** | | | **Amino acid change** | **Mutation-Type** |
| AIM1 | | | P597fs*47 | Deletion - Frameshift |
| KIAA0922 | | | W882fs*1 | Deletion - Frameshift |
| KIAA0922_ENST00000409959 | | | W1030fs*1 | Deletion - Frameshift |
| TCHHL1 | | | A554fs*5 | Deletion - Frameshift |
| ZNF317 | | | K584fs*>12 | Deletion - Frameshift |
| FGD5 | | | E632_E633delEE | Deletion - In frame |
| FGD5_ENST00000285046 | | | E873_E874delEE | Deletion - In frame |
| IRF2BPL | | | A161_A164delAAAA | Deletion - In frame |
| STK17B | | | S348delS | Deletion - In frame |
| USO1 | | | L632delL | Deletion - In frame |
| USO1_ENST00000538159 | | | L713delL | Deletion - In frame |
| CCDC42B | | | E187fs*116 | Insertion - Frameshift |
| KIF9 | | | S559fs*12 | Insertion - Frameshift |
| OR51B4 | | | Y3fs*2 | Insertion - Frameshift |
| AARSD1 | | | M1V | Substitution - Missense |
| ABCA4 | | | A192V | Substitution - Missense |
| ABCA4_ENST00000535735 | | | A192V | Substitution - Missense |
| ABCB6 | | | Y599C | Substitution - Missense |
| ABCB6_ENST00000265316 | | | Y599C | Substitution - Missense |
| ACLY | | | N807S | Substitution - Missense |
| ACP5 | | | N243S | Substitution - Missense |
| ACP5_ENST00000592828 | | | N243S | Substitution - Missense |
| ACTR10 | | | C141Y | Substitution - Missense |
| ACY3 | | | L113P | Substitution - Missense |
| ADAMTS1 | | | P133A | Substitution - Missense |
| ADAMTS14 | | | R252Q | Substitution - Missense |
| ADAMTS3 | | | E596Q | Substitution - Missense |
| ADAMTSL2 | | | P906L | Substitution - Missense |
| ADAMTSL2_ENST00000393061 | | | P1015L | Substitution - Missense |
| AGXT2 | | | M358K | Substitution - Missense |
| AKAP9 | | | Q2742R | Substitution - Missense |
| AKAP9_ENST00000356239 | | | Q2730R | Substitution - Missense |
| ALDH1L2 | | | V293I | Substitution - Missense |
| ALK | | | P158T | Substitution - Missense |
| ALK_ENST00000431873 | | | P158T | Substitution - Missense |
| ANK2 | | | R1191W | Substitution - Missense |
| ANK2_ENST00000506722 | | | R1182W | Substitution - Missense |
| ANK3 | | | P1708L | Substitution - Missense |
| ANKRD29 | | | D205H | Substitution - Missense |
| ANXA13 | | | Y332F | Substitution - Missense |
| ARHGEF26 | | | L108P | Substitution - Missense |
| ARHGEF26_ENST00000356448 | | | L108P | Substitution - Missense |
| ARHGEF26_ENST00000465817 | | | L108P | Substitution - Missense |
| ARID3B | | | K513N | Substitution - Missense |
| ARID5A | | | P556T | Substitution - Missense |
| ARID5A | | | P433A | Substitution - Missense |
| ARID5B | | | K1043R | Substitution - Missense |
| ARID5B_ENST00000309334 | | | K800R | Substitution - Missense |
| ARNT | | | P596L | Substitution - Missense |
| ATP5B | | | L95M | Substitution - Missense |
| ATP7B | | | D1296N | Substitution - Missense |
| ATP8B2 | | | T431N | Substitution - Missense |
| ATP8B2_ENST00000341822 | | | T417N | Substitution - Missense |
| ATP9B | | | A48V | Substitution - Missense |
| BCAS2 | | | N48S | Substitution - Missense |
| BIN3 | | | E109Q | Substitution - Missense |
| BMP1 | | | V542I | Substitution - Missense |
| BMP1_ENST00000306349 | | | V542I | Substitution - Missense |
| BMP1_ENST00000397814 | | | V542I | Substitution - Missense |
| BMP1_ENST00000397816 | | | V542I | Substitution - Missense |
| C10orf129_ENST00000341686 | | | S337N | Substitution - Missense |
| C10orf25 | | | R31W | Substitution - Missense |
| C10orf93_ENST00000368582 | | | G1496A | Substitution - Missense |
| C11orf41 | | | H56R | Substitution - Missense |
| C11orf41_ENST00000321505 | | | H56R | Substitution - Missense |
| C12orf50 | | | V250A | Substitution - Missense |
| C1orf124 | | | S400F | Substitution - Missense |
| C21orf13 | | | L609F | Substitution - Missense |
| C2orf71 | | | E136G | Substitution - Missense |
| C2orf71 | | | R29W | Substitution - Missense |
| C3orf25 | | | R338C | Substitution - Missense |
| C3orf30 | | | M198T | Substitution - Missense |
| CARNS1_ENST00000445895 | | | R208Q | Substitution - Missense |
| CCDC150 | | | L606F | Substitution - Missense |
| CDC42EP2 | | | K4R | Substitution - Missense |
| CDCA2 | | | R813G | Substitution - Missense |
| CDH5 | | | D301N | Substitution - Missense |
| CEP97 | | | L456S | Substitution - Missense |
| CEP97_ENST00000327230 | | | L456S | Substitution - Missense |
| CHFR | | | T554M | Substitution - Missense |
| CHFR_ENST00000432561 | | | T595M | Substitution - Missense |
| CIC | | | P1538L | Substitution - Missense |
| CNTRL_ENST00000373855 | | | R2233H | Substitution - Missense |
| COL17A1 | | | H74R | Substitution - Missense |
| COL28A1 | | | N941D | Substitution - Missense |
| COL6A2 | | | R1013C | Substitution - Missense |
| CRTC2 | | | M551K | Substitution - Missense |
| CYLC1 | | | E368Q | Substitution - Missense |
| CYLC1_ENST00000329312 | | | E368Q | Substitution - Missense |
| DCHS2 | | | Y639D | Substitution - Missense |
| DCHS2_ENST00000339452 | | | Y1138D | Substitution - Missense |
| DHX38 | | | D70A | Substitution - Missense |
| DISC1_ENST00000366633 | | | T561A | Substitution - Missense |
| DISC1_ENST00000366636 | | | T561A | Substitution - Missense |
| DISC1_ENST00000366638 | | | T593A | Substitution - Missense |
| DMBT1 | | | G2387D | Substitution - Missense |
| DMBT1_ENST00000368909 | | | G2387D | Substitution - Missense |
| DMBT1_ENST00000368915 | | | G2516D | Substitution - Missense |
| DMRTC2 | | | C344R | Substitution - Missense |
| DNAH17 | | | Y3861H | Substitution - Missense |
| DNAH17_ENST00000389840 | | | Y3852H | Substitution - Missense |
| DNAH3 | | | G1715S | Substitution - Missense |
| DNAH3_ENST00000261383 | | | G1715S | Substitution - Missense |
| DNAH6 | | | R380C | Substitution - Missense |
| DNAH6 | | | E2275K | Substitution - Missense |
| DNAH6_ENST00000398278 | | | R380C | Substitution - Missense |
| DNAH6_ENST00000398278 | | | E2275K | Substitution - Missense |
| DNAH8 | | | P4475L | Substitution - Missense |
| DNAH8_ENST00000359357 | | | P4475L | Substitution - Missense |
| DNM1L | | | T224P | Substitution - Missense |
| DNM1L_ENST00000553257 | | | T237P | Substitution - Missense |
| DSCAML1 | | | M1918I | Substitution - Missense |
| DYRK3 | | | R415H | Substitution - Missense |
| DYRK3_ENST00000367106 | | | R430H | Substitution - Missense |
| DYRK3_ENST00000367109 | | | R450H | Substitution - Missense |
| EGFL4 | | | V896I | Substitution - Missense |
| ELTD1 | | | T157A | Substitution - Missense |
| ENSG00000187812 | | | M13L | Substitution - Missense |
| EPB41L1 | | | R874T | Substitution - Missense |
| EPHA4 | | | S554L | Substitution - Missense |
| EPHA4_ENST00000281821 | | | S554L | Substitution - Missense |
| EPHA7 | | | A242V | Substitution - Missense |
| EPHA7_ENST00000369303 | | | A242V | Substitution - Missense |
| ERBB2 | | | T444S | Substitution - Missense |
| ERBB2_ENST00000541774 | | | T429S | Substitution - Missense |
| ERN2_ENST00000256797 | | | R204G | Substitution - Missense |
| EWSR1 | | | S260N | Substitution - Missense |
| EWSR1_ENST00000333395 | | | S260N | Substitution - Missense |
| EWSR1_ENST00000414183 | | | S266N | Substitution - Missense |
| EXD2 | | | R31C | Substitution - Missense |
| EXOC6 | | | S150R | Substitution - Missense |
| EXOC6_ENST00000371547 | | | S166R | Substitution - Missense |
| EXOC6_ENST00000371552 | | | S145R | Substitution - Missense |
| FAM135B | | | E53Q | Substitution - Missense |
| FAM184B | | | R281G | Substitution - Missense |
| FAM5C | | | R109C | Substitution - Missense |
| FAM63B | | | A56T | Substitution - Missense |
| FBN3 | | | R796Q | Substitution - Missense |
| FBXW10 | | | V736M | Substitution - Missense |
| FBXW10_ENST00000308799 | | | V765M | Substitution - Missense |
| FCER2 | | | R253W | Substitution - Missense |
| FHAD1_ENST00000358897 | | | E66K | Substitution - Missense |
| FHAD1_ENST00000375999 | | | E66K | Substitution - Missense |
| FOXN1 | | | P230R | Substitution - Missense |
| FREM1 | | | D1347V | Substitution - Missense |
| FRY | | | T2327A | Substitution - Missense |
| FUT7 | | | R243H | Substitution - Missense |
| FXYD5 | | | A49S | Substitution - Missense |
| FXYD5_ENST00000541435 | | | A49S | Substitution - Missense |
| GALR2 | | | R183G | Substitution - Missense |
| GAP43 | | | S131L | Substitution - Missense |
| GAP43_ENST00000393780 | | | S167L | Substitution - Missense |
| GBP2 | | | K460Q | Substitution - Missense |
| GBX1 | | | P234L | Substitution - Missense |
| GK2 | | | A2S | Substitution - Missense |
| GLA | | | R301Q | Substitution - Missense |
| GLIS2 | | | R236C | Substitution - Missense |
| GLYATL3 | | | Q99K | Substitution - Missense |
| GOLGA7 | | | Q98E | Substitution - Missense |
| GPR107 | | | E547K | Substitution - Missense |
| GPR107_ENST00000372406 | | | E595K | Substitution - Missense |
| GTF2E1 | | | S352N | Substitution - Missense |
| HEATR4 | | | E749K | Substitution - Missense |
| HEATR4_ENST00000553558 | | | E796K | Substitution - Missense |
| HGS | | | P357L | Substitution - Missense |
| HIP1 | | | E860Q | Substitution - Missense |
| HLA-DPB1 | | | T172A | Substitution - Missense |
| HLA-DPB1_ENST00000428835 | | | T149A | Substitution - Missense |
| IDUA | | | A344T | Substitution - Missense |
| IDUA_ENST00000453894 | | | A366T | Substitution - Missense |
| IL2RB | | | G398R | Substitution - Missense |
| INCENP | | | P815R | Substitution - Missense |
| INCENP_ENST00000394818 | | | P819R | Substitution - Missense |
| INTS9 | | | M519T | Substitution - Missense |
| INVS | | | L309V | Substitution - Missense |
| INVS_ENST00000541287 | | | L213V | Substitution - Missense |
| IPO4 | | | A1043T | Substitution - Missense |
| IPO9 | | | I80V | Substitution - Missense |
| IRS4 | | | D388N | Substitution - Missense |
| IRX6 | | | G383R | Substitution - Missense |
| JAK2 | | | V617F | Substitution - Missense |
| JMY_ENST00000396137 | | | V258A | Substitution - Missense |
| KCNG2 | | | D165Y | Substitution - Missense |
| KCNMA1_ENST00000428546 | | | A44D | Substitution - Missense |
| KIAA0284 | | | T1301I | Substitution - Missense |
| KIAA0284_ENST00000414716 | | | T1336I | Substitution - Missense |
| KIAA0649 | | | R381C | Substitution - Missense |
| KIAA0649 | | | T908R | Substitution - Missense |
| KIAA2026 | | | T434I | Substitution - Missense |
| KIAA2026_ENST00000399933 | | | T1259I | Substitution - Missense |
| KIF26A | | | R1761W | Substitution - Missense |
| KLF12 | | | P203S | Substitution - Missense |
| KLK3 | | | R250Q | Substitution - Missense |
| KNTC1 | | | F721Y | Substitution - Missense |
| KNTC1 | | | L1511F | Substitution - Missense |
| KRTAP10-11 | | | R269H | Substitution - Missense |
| KTN1_ENST00000416613 | | | T631A | Substitution - Missense |
| LAD1 | | | P319S | Substitution - Missense |
| LAD1_ENST00000367313 | | | P333S | Substitution - Missense |
| LAMB2 | | | R957Q | Substitution - Missense |
| LCA5L | | | L609F | Substitution - Missense |
| LMBRD2 | | | V572L | Substitution - Missense |
| LMTK3_ENST00000270238 | | | G659E | Substitution - Missense |
| LOC51059 | | | E53Q | Substitution - Missense |
| LOXL4 | | | R675Q | Substitution - Missense |
| LPA | | | P1269H | Substitution - Missense |
| LRRC42 | | | P395S | Substitution - Missense |
| LRRC46 | | | H41Y | Substitution - Missense |
| LRRC48 | | | I427T | Substitution - Missense |
| LRRC48_ENST00000411504 | | | I427T | Substitution - Missense |
| LTC4S | | | E4K | Substitution - Missense |
| LY9 | | | T253M | Substitution - Missense |
| MAGEB10 | | | I95M | Substitution - Missense |
| MAGEB10_ENST00000356790 | | | I95M | Substitution - Missense |
| MAGI1 | | | R77Q | Substitution - Missense |
| MAGI1_ENST00000330909 | | | R77Q | Substitution - Missense |
| MAGI1_ENST00000402939 | | | R77Q | Substitution - Missense |
| MAPK7 | | | A296T | Substitution - Missense |
| MAPKAPK5_ENST00000202788 | | | G445E | Substitution - Missense |
| MAPKAPK5_ENST00000551404 | | | G445E | Substitution - Missense |
| MAPKBP1 | | | S1112L | Substitution - Missense |
| MAPKBP1_ENST00000456763 | | | S1118L | Substitution - Missense |
| MCU | | | S72P | Substitution - Missense |
| MCU_ENST00000357157 | | | S72P | Substitution - Missense |
| MDFI | | | V80I | Substitution - Missense |
| MED17 | | | G74R | Substitution - Missense |
| MEGF8 | | | V1355I | Substitution - Missense |
| MEGF8_ENST00000334370 | | | V1288I | Substitution - Missense |
| MORN1 | | | H349R | Substitution - Missense |
| MRPS11 | | | R192W | Substitution - Missense |
| MUC5AC | | | T2041I | Substitution - Missense |
| MUC5B | | | T2038I | Substitution - Missense |
| MXRA5 | | | R2177G | Substitution - Missense |
| MXRA5_ENST00000381114 | | | R2177G | Substitution - Missense |
| MYBBP1A | | | T938A | Substitution - Missense |
| MYBBP1A_ENST00000381556 | | | T938A | Substitution - Missense |
| MYH6 | | | D629N | Substitution - Missense |
| N4BP2 | | | M1477V | Substitution - Missense |
| NEB | | | L3336P | Substitution - Missense |
| NEB_ENST00000397345 | | | L3579P | Substitution - Missense |
| NEB_ENST00000427231 | | | L3579P | Substitution - Missense |
| NIN | | | K877R | Substitution - Missense |
| NIN_ENST00000245441 | | | K877R | Substitution - Missense |
| NIN_ENST00000382041 | | | K877R | Substitution - Missense |
| NIN_ENST00000453196 | | | K877R | Substitution - Missense |
| NOTCH2 | | | A1972V | Substitution - Missense |
| NRIP1 | | | R85I | Substitution - Missense |
| NSMAF | | | V870I | Substitution - Missense |
| NSMAF_ENST00000427130 | | | V901I | Substitution - Missense |
| NT5E | | | V470I | Substitution - Missense |
| NYNRIN | | | S297R | Substitution - Missense |
| OR4A15 | | | A264T | Substitution - Missense |
| OR5AN1 | | | S268F | Substitution - Missense |
| OR5T3 | | | T108I | Substitution - Missense |
| OR9A2 | | | H12P | Substitution - Missense |
| OSBP2 | | | V397M | Substitution - Missense |
| OSBP2_ENST00000382310 | | | V397M | Substitution - Missense |
| OSBP2_ENST00000403222 | | | V232M | Substitution - Missense |
| OSBP2_ENST00000437268 | | | V139M | Substitution - Missense |
| PCDH15 | | | V1242M | Substitution - Missense |
| PCDH15 | | | V528I | Substitution - Missense |
| PCDH15_ENST00000395438 | | | V1242M | Substitution - Missense |
| PCDH15_ENST00000395438 | | | V528I | Substitution - Missense |
| PCDH15_ENST00000414778 | | | V1247M | Substitution - Missense |
| PCDH15_ENST00000414778 | | | V533I | Substitution - Missense |
| PCDH15_ENST00000417177 | | | V1247M | Substitution - Missense |
| PCDH15_ENST00000417177 | | | V533I | Substitution - Missense |
| PCDH18 | | | D995E | Substitution - Missense |
| PCM1 | | | S1323G | Substitution - Missense |
| PCNT | | | R1239H | Substitution - Missense |
| PEAR1 | | | P590L | Substitution - Missense |
| PEPD | | | R314H | Substitution - Missense |
| PER2 | | | I784M | Substitution - Missense |
| PFAS | | | R572Q | Substitution - Missense |
| PGAM4 | | | Y153H | Substitution - Missense |
| PHLPP2 | | | S18C | Substitution - Missense |
| PHLPP2_ENST00000360429 | | | S18C | Substitution - Missense |
| PIH1D2 | | | R57K | Substitution - Missense |
| PIK3C2B | | | I189T | Substitution - Missense |
| PLTP | | | V253M | Substitution - Missense |
| PLTP_ENST00000542937 | | | V273M | Substitution - Missense |
| POLQ | | | G236E | Substitution - Missense |
| POSTN | | | I724V | Substitution - Missense |
| PRAME | | | R13Q | Substitution - Missense |
| PSD3 | | | A469T | Substitution - Missense |
| PSD3_ENST00000440756 | | | A1003T | Substitution - Missense |
| PSMD13 | | | N143S | Substitution - Missense |
| PSMD13_ENST00000431206 | | | N145S | Substitution - Missense |
| PTGES3L | | | M62V | Substitution - Missense |
| PTGES3L-AARSD1 | | | M62V | Substitution - Missense |
| PXDNL_ENST00000356297 | | | C532W | Substitution - Missense |
| Q9BSM8_HUMAN | | | V138L | Substitution - Missense |
| Q9NT31_HUMAN | | | L52V | Substitution - Missense |
| RFX7 | | | K1254N | Substitution - Missense |
| RFX7_ENST00000423270 | | | K1351N | Substitution - Missense |
| RGS7BP | | | T236N | Substitution - Missense |
| RNF213 | | | A1541V | Substitution - Missense |
| RNF213_ENST00000411702 | | | A3517V | Substitution - Missense |
| RNH1 | | | R64C | Substitution - Missense |
| RPE65 | | | A145T | Substitution - Missense |
| RPTOR | | | P227L | Substitution - Missense |
| RPTOR_ENST00000537330 | | | P42L | Substitution - Missense |
| RRBP1_ENST00000377813 | | | Q408L | Substitution - Missense |
| RSAD1 | | | L351P | Substitution - Missense |
| SAA1 | | | W103R | Substitution - Missense |
| SCN3A | | | I1037T | Substitution - Missense |
| SCN3A_ENST00000409101 | | | I988T | Substitution - Missense |
| SDCCAG8 | | | T398M | Substitution - Missense |
| SDR9C7 | | | M158K | Substitution - Missense |
| SEMA3F | | | G113V | Substitution - Missense |
| SH2D3C_ENST00000373276 | | | G104R | Substitution - Missense |
| SIAH1 | | | R232Q | Substitution - Missense |
| SIAH1_ENST00000356721 | | | R263Q | Substitution - Missense |
| SIPA1 | | | N1013H | Substitution - Missense |
| SLC4A5 | | | T938M | Substitution - Missense |
| SLC4A5_ENST00000377634 | | | T938M | Substitution - Missense |
| SLC7A10 | | | V80F | Substitution - Missense |
| SLIT1 | | | R1460W | Substitution - Missense |
| SLIT2 | | | C1145R | Substitution - Missense |
| SMPD1 | | | H138R | Substitution - Missense |
| SMPD1_ENST00000342245 | | | H138R | Substitution - Missense |
| SPATA20 | | | V344I | Substitution - Missense |
| SPHK1 | | | D342G | Substitution - Missense |
| SPTBN5 | | | E2361K | Substitution - Missense |
| SPTLC3_ENST00000450297 | | | V191A | Substitution - Missense |
| SRGAP1_ENST00000357825 | | | R480Q | Substitution - Missense |
| SSX1 | | | Q98H | Substitution - Missense |
| STK16_ENST00000409638 | | | R112S | Substitution - Missense |
| SUSD1 | | | R416H | Substitution - Missense |
| SUSD1_ENST00000374264 | | | R416H | Substitution - Missense |
| SUSD2 | | | T47M | Substitution - Missense |
| TAAR5 | | | L278I | Substitution - Missense |
| TCEA1 | | | K4R | Substitution - Missense |
| TCEA1_ENST00000521604 | | | K188R | Substitution - Missense |
| TCF25 | | | A121S | Substitution - Missense |
| TM2D1 | | | Y28C | Substitution - Missense |
| TMCO6 | | | I166T | Substitution - Missense |
| TMEM123 | | | S69N | Substitution - Missense |
| TMEM132E | | | G858R | Substitution - Missense |
| TMEM86B | | | G173S | Substitution - Missense |
| TMPRSS11B | | | G206S | Substitution - Missense |
| TMPRSS15 | | | S13F | Substitution - Missense |
| TNR | | | Y396H | Substitution - Missense |
| TNR | | | G1001R | Substitution - Missense |
| TONSL | | | P747S | Substitution - Missense |
| TONSL_ENST00000409379 | | | P906S | Substitution - Missense |
| TP53 | | | R248W | Substitution - Missense |
| TP53_ENST00000269305 | | | R248W | Substitution - Missense |
| TP53_ENST00000413465 | | | R248W | Substitution - Missense |
| TP53_ENST00000420246 | | | R248W | Substitution - Missense |
| TP53_ENST00000455263 | | | R248W | Substitution - Missense |
| TP53_ENST00000545858 | | | R155W | Substitution - Missense |
| TRMT6 | | | P382A | Substitution - Missense |
| TSPYL6 | | | G319S | Substitution - Missense |
| TTC18 | | | P883T | Substitution - Missense |
| TTC19 | | | A469T | Substitution - Missense |
| TTC40 | | | G1496A | Substitution - Missense |
| TTK | | | D583N | Substitution - Missense |
| TTK_ENST00000369798 | | | D583N | Substitution - Missense |
| TTLL9 | | | H30L | Substitution - Missense |
| TTLL9_ENST00000375935 | | | H12L | Substitution - Missense |
| USP29 | | | L82F | Substitution - Missense |
| VIL1 | | | K374N | Substitution - Missense |
| VTA1 | | | R57C | Substitution - Missense |
| WDR87 | | | G276R | Substitution - Missense |
| WDR87_ENST00000447313 | | | G315R | Substitution - Missense |
| WISP3 | | | R5Q | Substitution - Missense |
| XAB2 | | | A381G | Substitution - Missense |
| ZAK | | | H651R | Substitution - Missense |
| ZDHHC20 | | | E302K | Substitution - Missense |
| ZDHHC20_ENST00000320220 | | | E302K | Substitution - Missense |
| ZDHHC20_ENST00000382466 | | | E302K | Substitution - Missense |
| ZFP36 | | | R254G | Substitution - Missense |
| ZFP36_ENST00000597629 | | | R260G | Substitution - Missense |
| ZMYND8 | | | E667D | Substitution - Missense |
| ZMYND8_ENST00000536340 | | | E674D | Substitution - Missense |
| ZNF274 | | | P412S | Substitution - Missense |
| ZNF274_ENST00000326804 | | | P444S | Substitution - Missense |
| ZNF280D | | | A788P | Substitution - Missense |
| ZNF318 | | | P1437S | Substitution - Missense |
| ZNF365_ENST00000395251 | | | I206M | Substitution - Missense |
| ZNF365_ENST00000410046 | | | I452M | Substitution - Missense |
| ZNF543 | | | C204Y | Substitution - Missense |
| ZNF579 | | | G215R | Substitution - Missense |
| ZNF774 | | | G35E | Substitution - Missense |
| ANO5 | | | W393* | Substitution - Nonsense |
| C19orf34 | | | R42* | Substitution - Nonsense |
| CTNND2 | | | Y172* | Substitution - Nonsense |
| MMP8 | | | G154* | Substitution - Nonsense |
| MX1 | | | K554* | Substitution - Nonsense |
| NOX3 | | | R227* | Substitution - Nonsense |
| RIC8A | | | Q440* | Substitution - Nonsense |
|  | | |  |  |
|  | | |  |  |
|  | | |  |  |
|  |  |  |  |  |
| **CbioPortal-CNV** | |  |  |  |
|  |  |  |  |  |
| **Gene** | **Expression** |  |  |  |
| CFHR3 | AMP |  |  |  |
| CFHR1 | AMP |  |  |  |
| OR2T10 | AMP |  |  |  |
| PRSS3P2 | DeepDel |  |  |  |
| PRSS2 | DeepDel |  |  |  |
| CDKN2A | DeepDel |  |  |  |
| CDKN2A-AS1 | DeepDel |  |  |  |
| UGT2B17 | DeepDel |  |  |  |
| CDKN2B | DeepDel |  |  |  |
| ACOT1 | DeepDel |  |  |  |
| CDKN2B-AS1 | DeepDel |  |  |  |
| MIR570 | AMP |  |  |  |
| CES1P1 | DeepDel |  |  |  |
| MTAP | DeepDel |  |  |  |
| SDHAP2 | AMP |  |  |  |
| LINC01193 | AMP |  |  |  |
| OR52N5 | DeepDel |  |  |  |
| DMRTA1 | DeepDel |  |  |  |
| LOC284344 | DeepDel |  |  |  |
| MIR-3648/3648 | AMP |  |  |  |
| MIR-365-2/365 | DeepDel |  |  |  |
| NBEAP1 | AMP |  |  |  |
| MIR31HG | DeepDel |  |  |  |
| MIR31 | DeepDel |  |  |  |
| LINC01239 | DeepDel |  |  |  |
| IFNE | DeepDel |  |  |  |
| POTEB | AMP |  |  |  |
| NF1P2 | AMP |  |  |  |
| IFNA1 | DeepDel |  |  |  |
| MST1L | AMP |  |  |  |
| IFNA8 | DeepDel |  |  |  |
| IFNA2 | DeepDel |  |  |  |
| IFNA13 | DeepDel |  |  |  |
| LOC646214 | AMP |  |  |  |
| KLHL9 | DeepDel |  |  |  |
| IFNA6 | DeepDel |  |  |  |
| CXADRP2 | AMP |  |  |  |
| IFNA5 | DeepDel |  |  |  |
| IFNA22P | DeepDel |  |  |  |
| ELAVL2 | DeepDel |  |  |  |
| IFNA14 | DeepDel |  |  |  |
| IFNA17 | DeepDel |  |  |  |
| IFNA7 | DeepDel |  |  |  |
| IFNA4 | DeepDel |  |  |  |
| IFNA10 | DeepDel |  |  |  |
| IFNA16 | DeepDel |  |  |  |
| IFNA21 | DeepDel |  |  |  |
| IFNW1 | DeepDel |  |  |  |
| GOLGA8CP | AMP |  |  |  |
| MIR-3653/3653 | AMP |  |  |  |
| IFNB1 | DeepDel |  |  |  |
| MIR-3163/3163 | DeepDel |  |  |  |
| GOLGA6L6 | AMP |  |  |  |
| HERC2P3 | AMP |  |  |  |
| HACD4 | DeepDel |  |  |  |
| FOCAD | DeepDel |  |  |  |
| MIR-3647/5P | DeepDel |  |  |  |
| MIR491 | DeepDel |  |  |  |
| MLLT3 | DeepDel |  |  |  |
| MIR4474 | DeepDel |  |  |  |
| TUSC1 | DeepDel |  |  |  |
| MIR4473 | DeepDel |  |  |  |
| RGPD4 | AMP |  |  |  |
| LOC100506422 | DeepDel |  |  |  |
| TYRP1 | DeepDel |  |  |  |
| OR4N3P | AMP |  |  |  |
| OR4M2 | AMP |  |  |  |
| OR4N4 | AMP |  |  |  |
| CAAP1 | DeepDel |  |  |  |
| PLAA | DeepDel |  |  |  |
| IFT74 | DeepDel |  |  |  |
| LRRC19 | DeepDel |  |  |  |
| TEK | DeepDel |  |  |  |
| LOC727924 | AMP |  |  |  |
| LINC00032 | DeepDel |  |  |  |
| EQTN | DeepDel |  |  |  |
| MOB3B | DeepDel |  |  |  |
| IFNK | DeepDel |  |  |  |
| C9ORF72 | DeepDel |  |  |  |
| MIR876 | DeepDel |  |  |  |
| MIR-3689A/3P | DeepDel |  |  |  |
| LINGO2 | DeepDel |  |  |  |
| MIR873 | DeepDel |  |  |  |
| LOC100506388 | DeepDel |  |  |  |
| ADPRHL1 | AMP |  |  |  |
| RGPD4-AS1 | AMP |  |  |  |
| F7 | AMP |  |  |  |
| F10 | AMP |  |  |  |
| PROZ | AMP |  |  |  |
| PCID2 | AMP |  |  |  |
| CUL4A | AMP |  |  |  |
| LAMP1 | AMP |  |  |  |
| GRTP1 | AMP |  |  |  |
| LIG4 | AMP |  |  |  |
| MYO16 | AMP |  |  |  |
| IRS2 | AMP |  |  |  |
| COL4A2 | AMP |  |  |  |
| RAB20 | AMP |  |  |  |
| CARKD | AMP |  |  |  |
| ARHGEF7 | AMP |  |  |  |
| MCF2L | AMP |  |  |  |
| ABHD13 | AMP |  |  |  |
| CARS2 | AMP |  |  |  |
| TEX29 | AMP |  |  |  |
| LINC00460 | AMP |  |  |  |
| TNFSF13B | AMP |  |  |  |
| COL4A1 | AMP |  |  |  |
| ING1 | AMP |  |  |  |
| MCF2L-AS1 | AMP |  |  |  |
| ANKRD10 | AMP |  |  |  |
| ATP11AUN | AMP |  |  |  |
| ATP11A | AMP |  |  |  |
| CCDC168 | AMP |  |  |  |
| TEX30 | AMP |  |  |  |
| KDELC1 | AMP |  |  |  |
| DAOA | AMP |  |  |  |
| EFNB2 | AMP |  |  |  |
| ARGLU1 | AMP |  |  |  |
| FAM155A | AMP |  |  |  |
| LINC00346 | AMP |  |  |  |
| DAOA-AS1 | AMP |  |  |  |
| SOX1 | AMP |  |  |  |
| TPP2 | AMP |  |  |  |
| METTL21C | AMP |  |  |  |
| BIVM | AMP |  |  |  |
| MIR-4776-1/5P | AMP |  |  |  |
| SPACA7 | AMP |  |  |  |
| TUBGCP3 | AMP |  |  |  |
| SLC10A2 | AMP |  |  |  |
| FGF14-AS2 | AMP |  |  |  |
| ITGBL1 | AMP |  |  |  |
| MIR4705 | AMP |  |  |  |
| ERCC5 | AMP |  |  |  |
| METTL21EP | AMP |  |  |  |
| FGF14-IT1 | AMP |  |  |  |
| BIVM-ERCC5 | AMP |  |  |  |
| FGF14 | AMP |  |  |  |
| MIR2681 | AMP |  |  |  |
| TFDP1 | DeepDel |  |  |  |
| CALHM2 | DeepDel |  |  |  |
| OR7E156P | AMP |  |  |  |
| PDCD1LG2 | AMP |  |  |  |
| ATP8B3 | DeepDel |  |  |  |
| SNORD95 | DeepDel |  |  |  |
| CD274 | AMP |  |  |  |
| MIR-3973/3973 | AMP |  |  |  |
| RLN1 | AMP |  |  |  |
| PLGRKT | AMP |  |  |  |
| RIC1 | AMP |  |  |  |
| INSL4 | AMP |  |  |  |
| RLN2 | AMP |  |  |  |
| ERMP1 | AMP |  |  |  |
| KIAA2026 | AMP |  |  |  |
| MLANA | AMP |  |  |  |
| MIR4665 | AMP |  |  |  |
| PSAPL1 | DeepDel |  |  |  |
| MIR1909 | DeepDel |  |  |  |
| LOC100288123 | DeepDel |  |  |  |
| RANBP6 | AMP |  |  |  |
| SNORD96A | DeepDel |  |  |  |
| REXO1 | DeepDel |  |  |  |
| TPD52L3 | AMP |  |  |  |
| ABLIM2 | DeepDel |  |  |  |
| DPP6 | DeepDel |  |  |  |
| IL33 | AMP |  |  |  |
| UHRF2 | AMP |  |  |  |
| SORCS2 | DeepDel |  |  |  |
| MIR4798 | DeepDel |  |  |  |
| MIR4274 | DeepDel |  |  |  |
| AFAP1 | DeepDel |  |  |  |
| DIP2A | AMP |  |  |  |
| MIR-3197/3197 | DeepDel |  |  |  |
| MIR-3193/3193 | DeepDel |  |  |  |
| YDJC | DeepDel |  |  |  |
| CLK4 | DeepDel |  |  |  |
| CYP2A7 | AMP |  |  |  |
| SIK1 | AMP |  |  |  |
| COL18A1 | AMP |  |  |  |
| SLC19A1 | AMP |  |  |  |
| AFAP1-AS1 | DeepDel |  |  |  |
| VIPR2 | DeepDel |  |  |  |
| ANKRD18A | DeepDel |  |  |  |
| RBM11 | AMP |  |  |  |
| NRIP1 | AMP |  |  |  |
| CCDC116 | DeepDel |  |  |  |
| GNB2L1 | DeepDel |  |  |  |
| COL6A1 | AMP |  |  |  |
| LSS | AMP |  |  |  |
| MCM3AP | AMP |  |  |  |
| C21ORF58 | AMP |  |  |  |
| PCNT | AMP |  |  |  |
| YBEY | AMP |  |  |  |
| MCM3AP-AS1 | AMP |  |  |  |
| COL18A1-AS1 | AMP |  |  |  |
| FAM201A | DeepDel |  |  |  |
| ABCC13 | AMP |  |  |  |
| SAMSN1 | AMP |  |  |  |
| PCBP3 | AMP |  |  |  |
| COL6A2 | AMP |  |  |  |
| FTCD | AMP |  |  |  |
| SPATC1L | AMP |  |  |  |
| LOC388813 | AMP |  |  |  |
| LOC100129027 | AMP |  |  |  |
| PAXIP1-AS2 | DeepDel |  |  |  |
| PAXIP1 | DeepDel |  |  |  |
| HTR5A | DeepDel |  |  |  |
| EN2 | DeepDel |  |  |  |
| SHH | DeepDel |  |  |  |
| CCL13 | DeepDel |  |  |  |
| HSPA13 | AMP |  |  |  |
| SLC25A1 | DeepDel |  |  |  |
| ADARB1 | AMP |  |  |  |
| LINC00315 | AMP |  |  |  |
| POFUT2 | AMP |  |  |  |
| LOC642852 | AMP |  |  |  |
| PAXIP1-AS1 | DeepDel |  |  |  |
| HTR5A-AS1 | DeepDel |  |  |  |
| LOC285889 | DeepDel |  |  |  |
| LINC00244 | DeepDel |  |  |  |
| LOC100506585 | DeepDel |  |  |  |
| ATP6V0E2 | DeepDel |  |  |  |
| CNPY1 | DeepDel |  |  |  |
| RBM33 | DeepDel |  |  |  |
| MNX1 | DeepDel |  |  |  |
| LINC00689 | DeepDel |  |  |  |
| ALDH1B1 | DeepDel |  |  |  |
| IGFBPL1 | DeepDel |  |  |  |
| TRAPPC10 | AMP |  |  |  |
| TRIM52 | DeepDel |  |  |  |
| SYK | DeepDel |  |  |  |
| KDM4B | DeepDel |  |  |  |
| DSCR8 | AMP |  |  |  |
| PDXK | AMP |  |  |  |
| CSTB | AMP |  |  |  |
| RRP1 | AMP |  |  |  |
| UBE2G2 | AMP |  |  |  |
| SUMO3 | AMP |  |  |  |
| PTTG1IP | AMP |  |  |  |
| ITGB2 | AMP |  |  |  |
| LINC01547 | AMP |  |  |  |
| FAM207A | AMP |  |  |  |
| LINC00163 | AMP |  |  |  |
| LINC00162 | AMP |  |  |  |
| AATBC | AMP |  |  |  |
| ATP6V0E2-AS1 | DeepDel |  |  |  |
| MNX1-AS1 | DeepDel |  |  |  |
| MIR153-2 | DeepDel |  |  |  |
| MIR-642B/642B | DeepDel |  |  |  |
| ITGB2-AS1 | AMP |  |  |  |
| CCHCR1 | DeepDel |  |  |  |
| C7ORF13 | DeepDel |  |  |  |
| RNF32 | DeepDel |  |  |  |
| NOM1 | DeepDel |  |  |  |
| UBE3C | DeepDel |  |  |  |
| DNAJB6 | DeepDel |  |  |  |
| PWP2 | AMP |  |  |  |
| CTSL3P | DeepDel |  |  |  |
| CBR3 | AMP |  |  |  |
| KCNJ15 | AMP |  |  |  |
| DSCR10 | AMP |  |  |  |
| LINC00114 | AMP |  |  |  |
| HMGN1 | AMP |  |  |  |
| RRP1B | AMP |  |  |  |
| C21ORF33 | AMP |  |  |  |
| TRPM2 | AMP |  |  |  |
| LRRC3 | AMP |  |  |  |
| TSPEAR | AMP |  |  |  |
| TSPEAR-AS2 | AMP |  |  |  |
| SSR4P1 | AMP |  |  |  |
| KRTAP12-2 | AMP |  |  |  |
| KRTAP12-1 | AMP |  |  |  |
| KRTAP10-10 | AMP |  |  |  |
| KRTAP10-4 | AMP |  |  |  |
| KRTAP10-6 | AMP |  |  |  |
| KRTAP10-7 | AMP |  |  |  |
| KRTAP10-9 | AMP |  |  |  |
| KRTAP10-1 | AMP |  |  |  |
| KRTAP10-11 | AMP |  |  |  |
| KRTAP10-2 | AMP |  |  |  |
| KRTAP10-5 | AMP |  |  |  |
| KRTAP10-8 | AMP |  |  |  |
| KRTAP10-3 | AMP |  |  |  |
| KRTAP12-3 | AMP |  |  |  |
| KRTAP12-4 | AMP |  |  |  |
| KRTAP10-12 | AMP |  |  |  |
| ZNF862 | DeepDel |  |  |  |
| INSIG1 | DeepDel |  |  |  |
| LMBR1 | DeepDel |  |  |  |
| AGPAT3 | AMP |  |  |  |
| UNC5A | DeepDel |  |  |  |
| CTSL | DeepDel |  |  |  |
| DOPEY2 | AMP |  |  |  |
| TTC3 | AMP |  |  |  |
| ERG | AMP |  |  |  |
| PSMG1 | AMP |  |  |  |
| BRWD1 | AMP |  |  |  |
| WRB | AMP |  |  |  |
| B3GALT5-AS1 | AMP |  |  |  |
| ICOSLG | AMP |  |  |  |
| DNMT3L | AMP |  |  |  |
| AIRE | AMP |  |  |  |
| PFKL | AMP |  |  |  |
| C21ORF2 | AMP |  |  |  |
| CBR3-AS1 | AMP |  |  |  |
| BRWD1-IT2 | AMP |  |  |  |
| PSORS1C2 | DeepDel |  |  |  |
| PTPRN2 | DeepDel |  |  |  |
| SLC25A51 | DeepDel |  |  |  |
| OR2V2 | DeepDel |  |  |  |
| DAPK1 | DeepDel |  |  |  |
| MORC3 | AMP |  |  |  |
| CHAF1B | AMP |  |  |  |
| CLDN14 | AMP |  |  |  |
| HLCS | AMP |  |  |  |
| RIPPLY3 | AMP |  |  |  |
| PIGP | AMP |  |  |  |
| DSCR9 | AMP |  |  |  |
| DSCR3 | AMP |  |  |  |
| DSCR4 | AMP |  |  |  |
| ETS2 | AMP |  |  |  |
| LCA5L | AMP |  |  |  |
| SH3BGR | AMP |  |  |  |
| SLC37A1 | AMP |  |  |  |
| POLR1E | DeepDel |  |  |  |
| SHB | DeepDel |  |  |  |
| HSPB3 | DeepDel |  |  |  |
| SNX18 | DeepDel |  |  |  |
| ZNF354A | DeepDel |  |  |  |
| AACSP1 | DeepDel |  |  |  |
| MIR4638 | DeepDel |  |  |  |
| TRIM41 | DeepDel |  |  |  |
| TRIM7 | DeepDel |  |  |  |
| ZCCHC7 | DeepDel |  |  |  |
| NMRK1 | DeepDel |  |  |  |
| OSTF1 | DeepDel |  |  |  |
| SETD4 | AMP |  |  |  |
| CBR1 | AMP |  |  |  |
| SIM2 | AMP |  |  |  |
| KCNJ6 | AMP |  |  |  |
| TFF1 | AMP |  |  |  |
| TMPRSS3 | AMP |  |  |  |
| UBASH3A | AMP |  |  |  |
| RSPH1 | AMP |  |  |  |
| U2AF1 | AMP |  |  |  |
| CRYAA | AMP |  |  |  |
| LINC00319 | AMP |  |  |  |
| HSF2BP | AMP |  |  |  |
| LOC100133286 | AMP |  |  |  |
| PRKAG2 | DeepDel |  |  |  |
| NCAPG2 | DeepDel |  |  |  |
| WDR60 | DeepDel |  |  |  |
| PAX5 | DeepDel |  |  |  |
| ZBTB5 | DeepDel |  |  |  |
| FBXO10 | DeepDel |  |  |  |
| TOMM5 | DeepDel |  |  |  |
| DCAF10 | DeepDel |  |  |  |
| HK3 | DeepDel |  |  |  |
| TSPAN17 | DeepDel |  |  |  |
| ZNF354B | DeepDel |  |  |  |
| ZFP2 | DeepDel |  |  |  |
| C9ORF40 | DeepDel |  |  |  |
| C9ORF41 | DeepDel |  |  |  |
| DYRK1A | AMP |  |  |  |
| B3GALT5 | AMP |  |  |  |
| TFF3 | AMP |  |  |  |
| TFF2 | AMP |  |  |  |
| PDE9A | AMP |  |  |  |
| NDUFV3 | AMP |  |  |  |
| PKNOX1 | AMP |  |  |  |
| CBS | AMP |  |  |  |
| LINC00313 | AMP |  |  |  |
| ZNF212 | DeepDel |  |  |  |
| ZNF783 | DeepDel |  |  |  |
| ZNF777 | DeepDel |  |  |  |
| ZNF746 | DeepDel |  |  |  |
| ZNF767P | DeepDel |  |  |  |
| KRBA1 | DeepDel |  |  |  |
| ZNF467 | DeepDel |  |  |  |
| SSPO | DeepDel |  |  |  |
| ESYT2 | DeepDel |  |  |  |
| FRMPD1 | DeepDel |  |  |  |
| TRMT10B | DeepDel |  |  |  |
| EXOSC3 | DeepDel |  |  |  |
| LINC00320 | AMP |  |  |  |
| HRH2 | DeepDel |  |  |  |
| ZNF454 | DeepDel |  |  |  |
| IGSF5 | AMP |  |  |  |
| C2CD2 | AMP |  |  |  |
| ZBTB21 | AMP |  |  |  |
| ABCG1 | AMP |  |  |  |
| WDR4 | AMP |  |  |  |
| EBLN3 | DeepDel |  |  |  |
| ZNF295-AS1 | AMP |  |  |  |
| LOC155060 | DeepDel |  |  |  |
| MIR595 | DeepDel |  |  |  |
| GRHPR | DeepDel |  |  |  |
| NCAM2 | AMP |  |  |  |
| GRM6 | DeepDel |  |  |  |
| ZNF879 | DeepDel |  |  |  |
| ZNF354C | DeepDel |  |  |  |
| TMPRSS2 | AMP |  |  |  |
| LINC00111 | AMP |  |  |  |
| LINC00479 | AMP |  |  |  |
| LINC00112 | AMP |  |  |  |
| PRDM15 | AMP |  |  |  |
| UMODL1 | AMP |  |  |  |
| UMODL1-AS1 | AMP |  |  |  |
| CD300C | DeepDel |  |  |  |
| HAR1A | DeepDel |  |  |  |
| HAR1B | DeepDel |  |  |  |
| MIR-649/649 | DeepDel |  |  |  |
| ADAMTS2 | DeepDel |  |  |  |
| MIR4476 | DeepDel |  |  |  |
| PCP4 | AMP |  |  |  |
| DSCAM | AMP |  |  |  |
| MIR4760 | AMP |  |  |  |
| LINC00323 | AMP |  |  |  |
| BACE2 | AMP |  |  |  |
| MIR3197 | AMP |  |  |  |
| FAM3B | AMP |  |  |  |
| MX2 | AMP |  |  |  |
| MX1 | AMP |  |  |  |
| RIPK4 | AMP |  |  |  |
| PLAC4 | AMP |  |  |  |
| DSCAM-AS1 | AMP |  |  |  |
| MBD3L2 | AMP |  |  |  |
| MBD3L3 | AMP |  |  |  |
| ZNF557 | AMP |  |  |  |
| INSR | AMP |  |  |  |
| FBN3 | AMP |  |  |  |
| CBY3 | DeepDel |  |  |  |
| MIR4475 | DeepDel |  |  |  |
| MIR4540 | DeepDel |  |  |  |
| GABBR2 | DeepDel |  |  |  |
| TNFSF15 | DeepDel |  |  |  |
| LOC100505478 | DeepDel |  |  |  |
| CCDC71L | DeepDel |  |  |  |
| MBD3L4 | AMP |  |  |  |
| MBD3L5 | AMP |  |  |  |
| MCOLN1 | AMP |  |  |  |
| PNPLA6 | AMP |  |  |  |
| STXBP2 | AMP |  |  |  |
| RETN | AMP |  |  |  |
| MCEMP1 | AMP |  |  |  |
| TRAPPC5 | AMP |  |  |  |
| ELAVL1 | AMP |  |  |  |
| CCL25 | AMP |  |  |  |
| KCNIP1 | DeepDel |  |  |  |
| RUFY1 | DeepDel |  |  |  |
| HNRNPH1 | DeepDel |  |  |  |
| OR2Y1 | DeepDel |  |  |  |
| ORM1 | DeepDel |  |  |  |
| ORM2 | DeepDel |  |  |  |
| AKNA | DeepDel |  |  |  |
| TNFSF8 | DeepDel |  |  |  |
| ARHGEF18 | AMP |  |  |  |
| PEX11G | AMP |  |  |  |
| C19ORF45 | AMP |  |  |  |
| ZNF358 | AMP |  |  |  |
| CAMSAP3 | AMP |  |  |  |
| XAB2 | AMP |  |  |  |
| PCP2 | AMP |  |  |  |
| PET100 | AMP |  |  |  |
| FCER2 | AMP |  |  |  |
| CLEC4G | AMP |  |  |  |
| MAP2K7 | AMP |  |  |  |
| TGFBR3L | AMP |  |  |  |
| SNAPC2 | AMP |  |  |  |
| CTXN1 | AMP |  |  |  |
| TIMM44 | AMP |  |  |  |
| C5ORF60 | DeepDel |  |  |  |
| CANX | DeepDel |  |  |  |
| MAML1 | DeepDel |  |  |  |
| LTC4S | DeepDel |  |  |  |
| MGAT4B | DeepDel |  |  |  |
| MIR1229 | DeepDel |  |  |  |
| SQSTM1 | DeepDel |  |  |  |
| C5ORF45 | DeepDel |  |  |  |
| TBC1D9B | DeepDel |  |  |  |
| RNF130 | DeepDel |  |  |  |
| MIR340 | DeepDel |  |  |  |
| RASGEF1C | DeepDel |  |  |  |
| MAPK9 | DeepDel |  |  |  |
| GFPT2 | DeepDel |  |  |  |
| CNOT6 | DeepDel |  |  |  |
| SCGB3A1 | DeepDel |  |  |  |
| FLT4 | DeepDel |  |  |  |
| GLIPR2 | DeepDel |  |  |  |
| CCIN | DeepDel |  |  |  |
| CLTA | DeepDel |  |  |  |
| GNE | DeepDel |  |  |  |
| RNF38 | DeepDel |  |  |  |
| MELK | DeepDel |  |  |  |
| RAD23B | DeepDel |  |  |  |
| KLF4 | DeepDel |  |  |  |
| COL27A1 | DeepDel |  |  |  |
| MIR455 | DeepDel |  |  |  |
| DFNB31 | DeepDel |  |  |  |
| ATP6V1G1 | DeepDel |  |  |  |
| C9ORF91 | DeepDel |  |  |  |
| LOC100128573 | AMP |  |  |  |
| MIR-493/493* | DeepDel |  |  |  |
| CDKN1B_PT157 | DeepDel |  |  |  |
| MAPK14_PT180 | DeepDel |  |  |  |
| BHLHA15 | DeepDel |  |  |  |
| TECPR1 | DeepDel |  |  |  |
| CD209 | AMP |  |  |  |
| CLEC4M | AMP |  |  |  |
| EVI5L | AMP |  |  |  |
| LRRC8E | AMP |  |  |  |
| GALNT10 | DeepDel |  |  |  |
| RECK | DeepDel |  |  |  |
| PAXBP1 | AMP |  |  |  |
| C21ORF62-AS1 | AMP |  |  |  |
| C17ORF77 | DeepDel |  |  |  |
| CD300E | DeepDel |  |  |  |
| CD300LD | DeepDel |  |  |  |
| CLEC4GP1 | AMP |  |  |  |
| PRR36 | AMP |  |  |  |
| LYPLA2P2 | AMP |  |  |  |
| BRI3 | DeepDel |  |  |  |
| BAIAP2L1 | DeepDel |  |  |  |
| PCDH1 | DeepDel |  |  |  |
| LOC729080 | DeepDel |  |  |  |
| NPTX2 | DeepDel |  |  |  |
| FAM166B | DeepDel |  |  |  |
| CD72 | DeepDel |  |  |  |
| MIR4667 | DeepDel |  |  |  |
| TESK1 | DeepDel |  |  |  |
| SIT1 | DeepDel |  |  |  |
| ARHGEF39 | DeepDel |  |  |  |
| CCDC107 | DeepDel |  |  |  |
| RMRP | DeepDel |  |  |  |
| CA9 | DeepDel |  |  |  |
| TPM2 | DeepDel |  |  |  |
| TLN1 | DeepDel |  |  |  |
| CREB3 | DeepDel |  |  |  |
| GBA2 | DeepDel |  |  |  |
| RGP1 | DeepDel |  |  |  |
| MSMP | DeepDel |  |  |  |
| NPR2 | DeepDel |  |  |  |
| SPAG8 | DeepDel |  |  |  |
| FAM221B | DeepDel |  |  |  |
| HINT2 | DeepDel |  |  |  |
| TMEM8B | DeepDel |  |  |  |
| OR13J1 | DeepDel |  |  |  |
| HRCT1 | DeepDel |  |  |  |
| LINC00961 | DeepDel |  |  |  |
| OR2S2 | DeepDel |  |  |  |
| LINC00950 | DeepDel |  |  |  |

**HEL Cell Definitions Kobayashi *et al.***

|  |  |  |
| --- | --- | --- |
| **Sanger-Mutation** | | |
|  |  |  |
| **Gene** | **Amino acid change** | **Mutation-Type** |
| C20orf194 | L117fs*16 | Deletion - Frameshift |
| CNKSR1 | P505fs*58 | Deletion - Frameshift |
| DSG4 | G400fs*5 | Deletion - Frameshift |
| DSG4_ENST00000359747 | G400fs*5 | Deletion - Frameshift |
| MCM3AP | P1267fs*3 | Deletion - Frameshift |
| SPAG17 | V1266fs*4 | Deletion - Frameshift |
| TIA1 | G284fs*7 | Deletion - Frameshift |
| NCOA6 | E1844delE | Deletion - In frame |
| PASK_ENST00000234040 | E1305fs*4 | Insertion - Frameshift |
| AACS | G610D | Substitution - Missense |
| AC003101.1 | G108S | Substitution - Missense |
| AC003101.1_ENST00000412403 | G108S | Substitution - Missense |
| AC007731_16 | T49M | Substitution - Missense |
| ACAD10 | R834H | Substitution - Missense |
| ACAD10_ENST00000455480 | R865H | Substitution - Missense |
| ACPL2 | T53M | Substitution - Missense |
| ACPL2_ENST00000393007 | T37M | Substitution - Missense |
| ADAM19 | P316R | Substitution - Missense |
| ADAM19_ENST00000257527 | P315R | Substitution - Missense |
| ADAM19_ENST00000394020 | P317R | Substitution - Missense |
| ADAM19_ENST00000430702 | P48R | Substitution - Missense |
| AHDC1 | S1058N | Substitution - Missense |
| AMZ2 | F151L | Substitution - Missense |
| ANKRD12 | R1113Q | Substitution - Missense |
| ANKRD30A | D1231Y | Substitution - Missense |
| ANKRD30A_ENST00000602533 | D1231Y | Substitution - Missense |
| APC | N862K | Substitution - Missense |
| ARHGEF12 | D308Y | Substitution - Missense |
| ATP7B | G869E | Substitution - Missense |
| BMP2 | G88A | Substitution - Missense |
| C14orf149 | R280Q | Substitution - Missense |
| C15orf2 | D304N | Substitution - Missense |
| C17orf28 | R164W | Substitution - Missense |
| C17orf80 | M488T | Substitution - Missense |
| C17orf80_ENST00000426147 | M488T | Substitution - Missense |
| C20orf114 | V203M | Substitution - Missense |
| C2orf16 | E832A | Substitution - Missense |
| C2orf16_ENST00000408964 | E832A | Substitution - Missense |
| C2orf54 | R115W | Substitution - Missense |
| C5 | L571V | Substitution - Missense |
| C6orf97 | S658L | Substitution - Missense |
| C9orf102_ENST00000407474 | K280E | Substitution - Missense |
| CASC3 | R350Q | Substitution - Missense |
| CASP9 | G402A | Substitution - Missense |
| CCDC12 | A93T | Substitution - Missense |
| CCDC12_ENST00000425441 | A106T | Substitution - Missense |
| CCDC41 | C221R | Substitution - Missense |
| CCDC90B | N171K | Substitution - Missense |
| CD244 | A52V | Substitution - Missense |
| CD244 | A52S | Substitution - Missense |
| CD244_ENST00000368032 | A52S | Substitution - Missense |
| CD244_ENST00000368032 | A52V | Substitution - Missense |
| CD244_ENST00000368033 | A52V | Substitution - Missense |
| CD244_ENST00000368033 | A52S | Substitution - Missense |
| CDC42BPA | A19S | Substitution - Missense |
| CDC42BPA_ENST00000334218 | A19S | Substitution - Missense |
| CDC42BPA_ENST00000366765 | A19S | Substitution - Missense |
| CDC42BPA_ENST00000366766 | A19S | Substitution - Missense |
| CDC42BPA_ENST00000366769 | A19S | Substitution - Missense |
| CDHR3 | T631P | Substitution - Missense |
| CDHR3_ENST00000317716 | T631P | Substitution - Missense |
| CDHR3_ENST00000542731 | T631P | Substitution - Missense |
| CDRT15L2 | A100V | Substitution - Missense |
| CHL1 | L427V | Substitution - Missense |
| CIDEA | E122D | Substitution - Missense |
| CIDEA_ENST00000320477 | E88D | Substitution - Missense |
| CLDND1 | R80W | Substitution - Missense |
| CLDND1_ENST00000437922 | R103W | Substitution - Missense |
| COL4A3 | A413T | Substitution - Missense |
| COL4A3_ENST00000328380 | A413T | Substitution - Missense |
| CORIN | S683R | Substitution - Missense |
| CYP27B1 | I198V | Substitution - Missense |
| CYP2A6 | Y351H | Substitution - Missense |
| DALRD3 | V357A | Substitution - Missense |
| DALRD3_ENST00000341949 | V524A | Substitution - Missense |
| DEGS1 | P266T | Substitution - Missense |
| DERA | G252A | Substitution - Missense |
| DKK1 | M178L | Substitution - Missense |
| DLD | S10F | Substitution - Missense |
| DLD_ENST00000205402 | S10F | Substitution - Missense |
| DNAH1 | R1087C | Substitution - Missense |
| DNAH1_ENST00000420323 | R1087C | Substitution - Missense |
| DTHD1 | L103V | Substitution - Missense |
| DTHD1_ENST00000456874 | L268V | Substitution - Missense |
| DUS3L | S563L | Substitution - Missense |
| EDAR | G226A | Substitution - Missense |
| EDAR_ENST00000409271 | G258A | Substitution - Missense |
| EGFL4 | R1673Q | Substitution - Missense |
| EIF2S3L | I140V | Substitution - Missense |
| ENG | H108Y | Substitution - Missense |
| ENG_ENST00000373203 | H108Y | Substitution - Missense |
| ENSG00000250305 | D383N | Substitution - Missense |
| EPB41L1 | R572C | Substitution - Missense |
| EPB41L1_ENST00000344237 | R861C | Substitution - Missense |
| EPX | R483H | Substitution - Missense |
| ERBB2IP | A1145V | Substitution - Missense |
| ERBB2IP_ENST00000284037 | A1145V | Substitution - Missense |
| ERBB2IP_ENST00000380943 | A1145V | Substitution - Missense |
| EXOSC4 | E238Q | Substitution - Missense |
| EXPH5 | Q1700R | Substitution - Missense |
| FAM111A | E267Q | Substitution - Missense |
| FAM135B | H1080Y | Substitution - Missense |
| FAM3D | S168F | Substitution - Missense |
| FAM63A | L186F | Substitution - Missense |
| FBN3 | R166C | Substitution - Missense |
| FCGRT | A294G | Substitution - Missense |
| FOXK1 | P214L | Substitution - Missense |
| GLI1 | G600C | Substitution - Missense |
| GLI4 | P14L | Substitution - Missense |
| HGD | E27A | Substitution - Missense |
| HIST1H2AD | P81L | Substitution - Missense |
| HOXB7 | G64R | Substitution - Missense |
| IGSF10 | S1873N | Substitution - Missense |
| IL28A | R161W | Substitution - Missense |
| IRAK3 | E494K | Substitution - Missense |
| JAK2 | V617F | Substitution - Missense |
| KIAA0415_ENST00000450194 | P40L | Substitution - Missense |
| KIAA1456 | D296N | Substitution - Missense |
| KIF7_ENST00000394412 | I464V | Substitution - Missense |
| KLK3 | R201L | Substitution - Missense |
| KLK3_ENST00000360617 | R201L | Substitution - Missense |
| KMT2D | T2254M | Substitution - Missense |
| KRT39 | L72V | Substitution - Missense |
| KRT40 | R159T | Substitution - Missense |
| KRTAP26-1 | S8L | Substitution - Missense |
| LCE1F | G65C | Substitution - Missense |
| LOC51059 | H1080Y | Substitution - Missense |
| LOC51321 | F209L | Substitution - Missense |
| LRRC37A3 | G1530E | Substitution - Missense |
| LRRC8C | G302R | Substitution - Missense |
| MADD | S700C | Substitution - Missense |
| MAN2B2 | N260S | Substitution - Missense |
| MAP3K5 | T1308I | Substitution - Missense |
| MAP3K7 | S328T | Substitution - Missense |
| MAP3K7_ENST00000369329 | S328T | Substitution - Missense |
| MB3L2_HUMAN | R201Q | Substitution - Missense |
| MCM3AP | S493N | Substitution - Missense |
| MEFV | M680I | Substitution - Missense |
| MEGF8 | R2132Q | Substitution - Missense |
| MEGF8_ENST00000334370 | R2065Q | Substitution - Missense |
| MLL2_ENST00000301067 | T2524M | Substitution - Missense |
| MLL4 | P174A | Substitution - Missense |
| MRS2 | G397A | Substitution - Missense |
| MRS2_ENST00000443868 | G400A | Substitution - Missense |
| MUC4 | F279Y | Substitution - Missense |
| MUC4 | F279I | Substitution - Missense |
| MUC4_ENST00000463781 | F4515Y | Substitution - Missense |
| MUC4_ENST00000463781 | F4515I | Substitution - Missense |
| MYO18B | R614W | Substitution - Missense |
| MYO1E | R362Q | Substitution - Missense |
| NBEAL2 | T1823M | Substitution - Missense |
| NBEAL2_ENST00000450053 | T2446M | Substitution - Missense |
| NLRP10 | E494D | Substitution - Missense |
| NR3C2 | R17K | Substitution - Missense |
| NUFIP1 | K402E | Substitution - Missense |
| OBFC1 | I327T | Substitution - Missense |
| OBSL1_ENST00000373873 | I592V | Substitution - Missense |
| OBSL1_ENST00000404537 | I592V | Substitution - Missense |
| OCIAD1 | P125R | Substitution - Missense |
| ODZ3 | R1765C | Substitution - Missense |
| OLR1 | L173F | Substitution - Missense |
| OPA1 | I24T | Substitution - Missense |
| OPTN | S269R | Substitution - Missense |
| OTOP1 | M495L | Substitution - Missense |
| PAK7 | M338L | Substitution - Missense |
| PCDHB4 | V662E | Substitution - Missense |
| PCM1 | S2010C | Substitution - Missense |
| PCSK2 | R605W | Substitution - Missense |
| PDK2 | E136K | Substitution - Missense |
| PDPN | I218V | Substitution - Missense |
| PHF3 | N99S | Substitution - Missense |
| PIK3R3 | L421P | Substitution - Missense |
| PLEKHG5_ENST00000377748 | P366S | Substitution - Missense |
| PLEKHG5_ENST00000535355 | P358S | Substitution - Missense |
| PLEKHG5_ENST00000537245 | P368S | Substitution - Missense |
| POLE | I634V | Substitution - Missense |
| POLE_ENST00000320574 | I634V | Substitution - Missense |
| POLRMT | G327E | Substitution - Missense |
| POLRMT_ENST00000588649 | G342E | Substitution - Missense |
| PRKCE | P507A | Substitution - Missense |
| PTPN9 | R332C | Substitution - Missense |
| PWP2 | Y182H | Substitution - Missense |
| PYCR2 | V92M | Substitution - Missense |
| Q5I0X0_HUMAN | I140V | Substitution - Missense |
| Q86YR2_HUMAN | K84T | Substitution - Missense |
| RBMX | S249Y | Substitution - Missense |
| RBMX_ENST00000431446 | P141T | Substitution - Missense |
| RGR_ENST00000372092 | W119R | Substitution - Missense |
| RINL | R401G | Substitution - Missense |
| RINL_ENST00000591812 | R515G | Substitution - Missense |
| RNF207 | E593Q | Substitution - Missense |
| RP11-1280I22.1 | R163Q | Substitution - Missense |
| RP11-1280I22.1_ENST00000302125 | R163Q | Substitution - Missense |
| RPL32 | A3S | Substitution - Missense |
| RPL7 | N80Y | Substitution - Missense |
| SC4MOL | N236S | Substitution - Missense |
| SCN11A | G798R | Substitution - Missense |
| SENP7 | M445V | Substitution - Missense |
| SENP7_ENST00000394095 | M511V | Substitution - Missense |
| SGSH | R169W | Substitution - Missense |
| SGSM2 | G411R | Substitution - Missense |
| SLC36A3 | A158V | Substitution - Missense |
| SLC4A7 | R318H | Substitution - Missense |
| SLITRK5 | C882S | Substitution - Missense |
| SLITRK5_ENST00000400028 | C641S | Substitution - Missense |
| SOHLH2 | T314M | Substitution - Missense |
| STRA6 | S112N | Substitution - Missense |
| STRA6_ENST00000563965 | S151N | Substitution - Missense |
| SULT1C2 | L138I | Substitution - Missense |
| SULT1C2 | F137L | Substitution - Missense |
| SULT1C2_ENST00000251481 | L127I | Substitution - Missense |
| SULT1C2_ENST00000251481 | F126L | Substitution - Missense |
| SYNJ2 | T1390A | Substitution - Missense |
| TAS2R13 | L109H | Substitution - Missense |
| TBX15 | L304W | Substitution - Missense |
| TBX15_ENST00000369429 | L410W | Substitution - Missense |
| TBX15_ENST00000449873 | L138W | Substitution - Missense |
| TCIRG1 | P390S | Substitution - Missense |
| TET2 | P1575L | Substitution - Missense |
| TFB2M | T372I | Substitution - Missense |
| TGOLN2_ENST00000377386 | R259G | Substitution - Missense |
| THAP10 | R132H | Substitution - Missense |
| THNSL2 | A10T | Substitution - Missense |
| THNSL2_ENST00000343544 | A10T | Substitution - Missense |
| TMEM132C | V311L | Substitution - Missense |
| TMEM132C_ENST00000435159 | V695L | Substitution - Missense |
| TMEM200C | D528E | Substitution - Missense |
| TNXB_ENST00000375244 | R847Q | Substitution - Missense |
| TNXB_ENST00000375247 | R799Q | Substitution - Missense |
| TP53 | M133K | Substitution - Missense |
| TP53_ENST00000269305 | M133K | Substitution - Missense |
| TP53_ENST00000413465 | M133K | Substitution - Missense |
| TP53_ENST00000414315 | M1K | Substitution - Missense |
| TP53_ENST00000420246 | M133K | Substitution - Missense |
| TP53_ENST00000455263 | M133K | Substitution - Missense |
| TP53_ENST00000545858 | M40K | Substitution - Missense |
| TP63 | E636Q | Substitution - Missense |
| TP63_ENST00000354600 | E542Q | Substitution - Missense |
| TRIM14 | G113E | Substitution - Missense |
| TSTA3 | P125L | Substitution - Missense |
| TTC12 | L613V | Substitution - Missense |
| TTC12_ENST00000314756 | L613V | Substitution - Missense |
| TTC39A_ENST00000262676 | G368R | Substitution - Missense |
| TTN_ENST00000342175 | V17842A | Substitution - Missense |
| TTN_ENST00000342992 | V24147A | Substitution - Missense |
| TTN_ENST00000356127 | V24145A | Substitution - Missense |
| TTN_ENST00000359218 | V17775A | Substitution - Missense |
| TUBGCP6 | N1186S | Substitution - Missense |
| TXNRD3 | R425C | Substitution - Missense |
| UBE4A | Q39L | Substitution - Missense |
| UNC5D | S221A | Substitution - Missense |
| UNC5D_ENST00000404895 | S226A | Substitution - Missense |
| USH2A | Y4318F | Substitution - Missense |
| USH2A_ENST00000366943 | Y4318F | Substitution - Missense |
| USP17L2 | L526P | Substitution - Missense |
| USP44 | S496P | Substitution - Missense |
| VCAN | V955D | Substitution - Missense |
| VPS13B | Y1164H | Substitution - Missense |
| VPS13B_ENST00000357162 | Y1164H | Substitution - Missense |
| VWF | T2023M | Substitution - Missense |
| WDR73 | D118E | Substitution - Missense |
| ZDHHC5 | V181I | Substitution - Missense |
| ZFHX2 | D2061N | Substitution - Missense |
| ZNF229 | T791N | Substitution - Missense |
| ZNF251 | T413A | Substitution - Missense |
| ZNF251_ENST00000292562 | T456A | Substitution - Missense |
| ZNF568 | S204N | Substitution - Missense |
| ZNF703 | G558S | Substitution - Missense |
| ZNF737 | T522S | Substitution - Missense |
| ZNF737_ENST00000427401 | T523S | Substitution - Missense |
| ZSCAN5A | D444A | Substitution - Missense |
| CEP164 | K318* | Substitution - Nonsense |
| CMAHP | R153* | Substitution - Nonsense |
| CMAHP_ENST00000377993 | R153* | Substitution - Nonsense |
|  |  |  |

|  |  |  |
| --- | --- | --- |
| **cBioPortal-Mutation** | | |
|  |  |  |
| **Gene** | **Amino acid change** | **Mutation-Type** |
| CTBP2 | Frame_Shift_Del | K974fs |
| RECQL4 | Frame_Shift_Del | R766fs |
| MLL3 | Frame_Shift_Ins | Y816fs |
| PASK | Frame_Shift_Ins | G1311fs |
| PRKDC | Frame_Shift_Ins | L1244fs |
| AAK1 | In_Frame_Del | 541_542QQ>Q |
| ALPK2 | In_Frame_Del | 1388_1389FF>F |
| CHD1 | In_Frame_Del | P1684del |
| GPR112 | In_Frame_Del | D2657del |
| MAML2 | In_Frame_Del | 604_607QQQQ>Q |
| MSH3 | In_Frame_Del | AAAAAAAPP56del |
| MSH3 | In_Frame_Del | PPA66del |
| MYST4 | In_Frame_Del | E1104del |
| NCOA3 | In_Frame_Del | Q1276del |
| TNRC6B | In_Frame_Del | Q1328del |
| AKAP9 | In_Frame_Ins | 1335_1336insQ |
| NR1H2 | In_Frame_Ins | 176_177insQ |
| AMZ2 | Missense_Mutation | F209L |
| APC | Missense_Mutation | N862K |
| ARHGEF12 | Missense_Mutation | D308Y |
| C15orf2 | Missense_Mutation | D304N |
| C6orf97 | Missense_Mutation | S658L |
| CASP9 | Missense_Mutation | G402A |
| CDC42BPA | Missense_Mutation | A19S |
| CHL1 | Missense_Mutation | L427V |
| CYP27B1 | Missense_Mutation | I198V |
| JAK2 | Missense_Mutation | V617F |
| KLK3 | Missense_Mutation | R201L |
| LRP2 | Missense_Mutation | T2086S |
| MAP3K7 | Missense_Mutation | S328T |
| MCM3AP | Missense_Mutation | S493N |
| PCM1 | Missense_Mutation | S2010C |
| PHF3 | Missense_Mutation | N99S |
| PRKCE | Missense_Mutation | P507A |
| TP53 | Missense_Mutation | M133K |
| TP63 | Missense_Mutation | E636Q |
| TTN | Missense_Mutation | V24147A |
| VPS13B | Missense_Mutation | Y1164H |
| MAML3 | Splice_Site_Del | Q767_splice |
| VEGFC | Splice_Site_Del | S419_splice |
| ASPH | Splice_Site_Ins | A264_splice |
| CLTCL1 | Splice_Site_Ins | V1201_splice |
| GRIA3 | Splice_Site_Ins |  |
| ITPR2 | Splice_Site_Ins | R470_splice |
| NEK3 | Splice_Site_Ins | K313_splice |
| PPP3R1 | Splice_Site_SNP | F15_splice |
| RNASEL | Splice_Site_SNP | I636_splice |
| PRKD3 | Stop_Codon_Ins |  |

|  |  |
| --- | --- |
| **Sanger-CNV** | |
|  |  |
| **Gene** | **Expression** |
| AK3 | Gain |
| C9orf150 | Gain |
| C9orf46 | Gain |
| C9orf68 | Gain |
| CD274 | Gain |
| CDC37L1 | Gain |
| ERMP1 | Gain |
| ERMP1_ENST00000543230 | Gain |
| INSL4 | Gain |
| INSL6 | Gain |
| JAK2 | Gain |
| KIAA1432 | Gain |
| KIAA1432_ENST00000381532 | Gain |
| KIAA2026 | Gain |
| KIAA2026_ENST00000399933 | Gain |
| MLANA | Gain |
| MPDZ | Gain |
| MPDZ_ENST00000319217 | Gain |
| MPDZ_ENST00000541718 | Gain |
| PDCD1LG2 | Gain |
| PPAPDC2 | Gain |
| RANBP6 | Gain |
| RCL1 | Gain |
| RCL1_ENST00000381732 | Gain |
| RLN1 | Gain |
| RLN2 | Gain |
| SLC1A1 | Gain |
| SPATA6L | Gain |
| TUSC1 | Gain |
| TYRP1 | Gain |
| AMELY | Loss |
| BCORP1 | Loss |
| C9orf53 | Loss |
| C9orf66 | Loss |
| CBWD1 | Loss |
| CBWD1_ENST00000314367 | Loss |
| CBWD1_ENST00000377447 | Loss |
| CDKN2A | Loss |
| CDKN2A_ENST00000361570 | Loss |
| CDKN2A_ENST00000446177 | Loss |
| CDKN2A_ENST00000498124 | Loss |
| CDKN2B | Loss |
| CDKN2B_ENST00000380142 | Loss |
| CDY1B | Loss |
| CDY2A | Loss |
| CDY2B | Loss |
| CYorf15B | Loss |
| CYorf17 | Loss |
| DAZ1 | Loss |
| DAZ1_ENST00000382510 | Loss |
| DAZ2 | Loss |
| DDX3Y | Loss |
| DMRT1 | Loss |
| DMRT2 | Loss |
| DMRT2_ENST00000302441 | Loss |
| DMRT3 | Loss |
| DMRT3_ENST00000417254 | Loss |
| DMRTA1 | Loss |
| DOCK8 | Loss |
| DOCK8_ENST00000453981 | Loss |
| EIF1AY | Loss |
| ELAVL2 | Loss |
| ELAVL2_ENST00000359598 | Loss |
| ENSG00000183704 | Loss |
| ENSG00000196076 | Loss |
| ENSG00000225516 | Loss |
| FAM106A | Loss |
| FAM197Y1 | Loss |
| FOXD4 | Loss |
| FOXD4_ENST00000382500 | Loss |
| GYG2P1 | Loss |
| HEATR2 | Loss |
| HEATR2_ENST00000313147 | Loss |
| HSFY1 | Loss |
| HSFY2 | Loss |
| IFNA1 | Loss |
| IFNA10 | Loss |
| IFNA13 | Loss |
| IFNA14 | Loss |
| IFNA16 | Loss |
| IFNA17 | Loss |
| IFNA2 | Loss |
| IFNA21 | Loss |
| IFNA4 | Loss |
| IFNA5 | Loss |
| IFNA6 | Loss |
| IFNA7 | Loss |
| IFNA8 | Loss |
| IFNB1 | Loss |
| IFNE | Loss |
| IFNW1 | Loss |
| IZUMO3 | Loss |
| KANK1 | Loss |
| KANK1_ENST00000354485 | Loss |
| KANK1_ENST00000382286 | Loss |
| KANK1_ENST00000382303 | Loss |
| KDM5D | Loss |
| KIAA1797 | Loss |
| KLHL9 | Loss |
| LGALS9C | Loss |
| MEF2C | Loss |
| MEF2C_ENST00000424173 | Loss |
| MEF2C_ENST00000437473 | Loss |
| MEF2C_ENST00000504921 | Loss |
| MTAP | Loss |
| NLGN4Y | Loss |
| NLGN4Y_ENST00000297967 | Loss |
| NLGN4Y_ENST00000382868 | Loss |
| PCDH11Y | Loss |
| PCDH11Y_ENST00000215473 | Loss |
| PCDH11Y_ENST00000215473 | Loss |
| PCDH11Y_ENST00000215473 | Loss |
| PCDH11Y_ENST00000362095 | Loss |
| PRKY | Loss |
| PRY | Loss |
| PRY2 | Loss |
| PTPLAD2 | Loss |
| Q8WTY6_HUMAN | Loss |
| RBMY1D_ENST00000382680 | Loss |
| RBMY1E | Loss |
| RBMY1F | Loss |
| RBMY1J | Loss |
| RPS4Y1 | Loss |
| RPS4Y1_ENST00000430575 | Loss |
| RPS4Y2 | Loss |
| SCAPER | Loss |
| SCAPER_ENST00000538941 | Loss |
| SIRPB1 | Loss |
| SIRPB1_ENST00000279477 | Loss |
| SRY | Loss |
| TBL1Y | Loss |
| TGIF2LY | Loss |
| TMSB4Y | Loss |
| TSPY1 | Loss |
| TSPY10 | Loss |
| TSPY3 | Loss |
| TSPY3_ENST00000383010 | Loss |
| TSPY3_ENST00000457222 | Loss |
| TSPY4 | Loss |
| TSPY8 | Loss |
| TSPY8_ENST00000287721 | Loss |
| TXLNG2P | Loss |
| USP9Y | Loss |
| UTY | Loss |
| UTY_ENST00000329134 | Loss |
| UTY_ENST00000331397 | Loss |
| UTY_ENST00000382896 | Loss |
| UTY_ENST00000540140 | Loss |
| VCY | Loss |
| VCY1B | Loss |
| ZFY | Loss |
|  |  |

|  |  |
| --- | --- |
| **CbioPortal-CNV** | |
|  |  |
| **Gene** | **Expression** |
| ADAM6 | AMP |
| KIAA0125 | AMP |
| OR2T10 | AMP |
| OR2T11 | AMP |
| PRSS3P2 | DeepDel |
| PRSS2 | DeepDel |
| GSTT1 | AMP |
| LOC391322 | AMP |
| CDKN2A | DeepDel |
| OR2T34 | AMP |
| CDKN2A-AS1 | DeepDel |
| LRRC37A2 | AMP |
| CDKN2B | DeepDel |
| MIR-3157/5P | DeepDel |
| ARL17A | AMP |
| LGALS9C | DeepDel |
| LRRC37A | AMP |
| CDKN2B-AS1 | DeepDel |
| NSFP1 | AMP |
| ARL17B | AMP |
| MIR570 | AMP |
| GSTTP2 | AMP |
| USP32P2 | DeepDel |
| FAM106A | DeepDel |
| OR4F5 | DeepDel |
| WASH7P | DeepDel |
| KANSL1-AS1 | AMP |
| DDX11L9 | AMP |
| FAM138E | AMP |
| OR4F4 | AMP |
| WASH3P | AMP |
| MIR-4750/4750 | AMP |
| OR4N4 | DeepDel |
| OR4M2 | DeepDel |
| MIR-3656/3656 | DeepDel |
| OR4N3P | DeepDel |
| MTAP | DeepDel |
| TBC1D3B | DeepDel |
| REREP3 | DeepDel |
| ANKRD30BP2 | AMP |
| GSTTP1 | AMP |
| CCL3L1 | DeepDel |
| CCL3L3 | DeepDel |
| OR2T29 | AMP |
| KIR2DL4 | AMP |
| DMRTA1 | DeepDel |
| DUSP22 | DeepDel |
| KRT17P5 | DeepDel |
| MIR-365-2/365 | DeepDel |
| MIR31HG | DeepDel |
| MIR31 | DeepDel |
| LINC01239 | DeepDel |
| OR2T35 | AMP |
| IFNE | DeepDel |
| IFNA1 | DeepDel |
| MST1L | AMP |
| MIR-3614/5P | AMP |
| KIR3DL1 | AMP |
| IFNA8 | DeepDel |
| IFNA2 | DeepDel |
| IFNA13 | DeepDel |
| KLHL9 | DeepDel |
| IFNA6 | DeepDel |
| CCDC144B | DeepDel |
| TP53TG3B | DeepDel |
| TP53TG3C | DeepDel |
| TP53TG3 | DeepDel |
| KIR2DS4 | AMP |
| IFNA5 | DeepDel |
| TBC1D3C | DeepDel |
| IFNA22P | DeepDel |
| TAS2R43 | AMP |
| ELAVL2 | DeepDel |
| LOC390705 | DeepDel |
| IFNA14 | DeepDel |
| IFNA17 | DeepDel |
| IFNA7 | DeepDel |
| SLC6A10P | DeepDel |
| IFNA4 | DeepDel |
| IFNA10 | DeepDel |
| IFNA16 | DeepDel |
| UGT2B28 | DeepDel |
| IFNA21 | DeepDel |
| IFNW1 | DeepDel |
| IFNB1 | DeepDel |
| SLC7A5P2 | DeepDel |
| HACD4 | DeepDel |
| KIR2DL1 | AMP |
| LOC729737 | DeepDel |
| ESPNP | AMP |
| CABLES2 | AMP |
| RBBP8NL | AMP |
| ADRM1 | AMP |
| RPS21 | AMP |
| OSBPL2 | AMP |
| LAMA5 | AMP |
| MIR4758 | AMP |
| TMPRSS11E | DeepDel |
| HRH3 | AMP |
| DOCK8 | DeepDel |
| TAF4 | AMP |
| LSM14B | AMP |
| PSMA7 | AMP |
| SS18L1 | AMP |
| MTG2 | AMP |
| MIR-4442/4442 | AMP |
| CDH4 | AMP |
| MIR1257 | AMP |
| MIR-4441/4441 | AMP |
| KIR2DL3 | AMP |
| C9ORF66 | DeepDel |
| KANK1 | DeepDel |
| DMRT1 | DeepDel |
| DMRT2 | DeepDel |
| DMRT3 | DeepDel |
| GSTM1 | AMP |
| FAM138C | DeepDel |
| WASH1 | DeepDel |
| MIR-378/378 | AMP |
| CBWD1 | DeepDel |
| MIR-3616/5P | AMP |
| FOXD4 | DeepDel |
| NPIPB3 | DeepDel |
| LOC100190986 | DeepDel |
| SMG1P3 | DeepDel |
| BCL2L1 | AMP |
| COX4I2 | AMP |
| TPX2 | AMP |
| ERVV-1 | AMP |
| MIR-370/370 | AMP |
| ID1 | AMP |
| MIR3193 | AMP |
| MYLK2 | AMP |
| MIR-3690/3690 | AMP |
| MCTS2P | AMP |
| REM1 | AMP |
| LINC00028 | AMP |
| HM13 | AMP |
| FOXS1 | AMP |
| DUSP15 | AMP |
| DEFB116 | AMP |
| DEFB124 | AMP |
| TTLL9 | AMP |
| DEFB123 | AMP |
| PDRG1 | AMP |
| DEFB115 | AMP |
| DEFB118 | AMP |
| DEFB119 | AMP |
| DEFB122 | AMP |
| XKR7 | AMP |
| CCM2L | AMP |
| DEFB121 | AMP |
| HCK | AMP |
| TM9SF4 | AMP |
| ERVV-2 | AMP |
| MIR-4723/3P | AMP |
| TSPY26P | AMP |
| PLAGL2 | AMP |
| MIR644A | AMP |
| MIR-376A-1/376A | AMP |
| POFUT1 | AMP |
| AHCY | AMP |
| ITCH | AMP |
| DYNLRB1 | AMP |
| PIGU | AMP |
| TP53INP2 | AMP |
| KIF3B | AMP |
| CHMP4B | AMP |
| ASIP | AMP |
| MAP1LC3A | AMP |
| NCOA6 | AMP |
| GGT7 | AMP |
| TRPC4AP | AMP |
| HMGB3P1 | AMP |
| MIR-3647/5P | AMP |
| OR4F13P | AMP |
| ASXL1 | AMP |
| ACSS2 | AMP |
| GSS | AMP |
| MYH7B | AMP |
| MIR499A | AMP |
| CEP250 | AMP |
| MIR499B | AMP |
| MIR-377/377* | AMP |
| E2F1 | AMP |
| PXMP4 | AMP |
| RALY | AMP |
| EDEM2 | AMP |
| PROCR | AMP |
| GDF5 | AMP |
| C20ORF173 | AMP |
| NECAB3 | AMP |
| C20ORF144 | AMP |
| ACTL10 | AMP |
| MIR4755 | AMP |
| EIF2S2 | AMP |
| MMP24 | AMP |
| EIF6 | AMP |
| FAM83C | AMP |
| UQCC1 | AMP |
| BPIFA4P | AMP |
| BPIFA3 | AMP |
| BPIFB1 | AMP |
| ZNF341 | AMP |
| ERGIC3 | AMP |
| BPIFA2 | AMP |
| BPIFA1 | AMP |
| CDK5RAP1 | AMP |
| CBFA2T2 | AMP |
| PHF20 | AMP |
| CNBD2 | AMP |
| OR4F15 | AMP |
| OR4F6 | AMP |
| SUN5 | AMP |
| BPIFB3 | AMP |
| BPIFB4 | AMP |
| SCAND1 | AMP |
| LBP | AMP |
| MIR-320D-1/320D | AMP |
| COMMD7 | AMP |
| BPIFB2 | AMP |
| BPIFB6 | AMP |
| SNTA1 | AMP |
| FER1L4 | AMP |
| BPI | AMP |
| SNHG17 | AMP |
| SNORA71B | AMP |
| SNORA71A | AMP |
| SNORA71C | AMP |
| SNORA71D | AMP |
| SNHG11 | AMP |
| SNORA60 | AMP |
| SNORA71E | AMP |
| SPATA41 | AMP |
| DNMT3B | AMP |
| MAPRE1 | AMP |
| NFS1 | AMP |
| RBM39 | AMP |
| LINC00657 | AMP |
| TGM2 | AMP |
| KIAA1755 | AMP |
| CERS3 | AMP |
| TARSL2 | AMP |
| TM2D3 | AMP |
| SPAG4 | AMP |
| CPNE1 | AMP |
| RBM12 | AMP |
| ROMO1 | AMP |
| EPB41L1 | AMP |
| AAR2 | AMP |
| RPRD1B | AMP |
| ADAMTS17 | AMP |
| LINS | AMP |
| ASB7 | AMP |
| ALDH1A3 | AMP |
| VIMP | AMP |
| SNRPA1 | AMP |
| DLGAP4 | AMP |
| TTI1 | AMP |
| TRAPPC2L | DeepDel |
| MIR-320E/320E | AMP |
| LOC100507472 | AMP |
| MIR-518A-2/3P | AMP |
| DNM1P46 | AMP |
| LRRK1 | AMP |
| PCSK6 | AMP |
| RALGAPB | AMP |
| PRSS45 | AMP |
| SYNM | AMP |
| TTC23 | AMP |
| LRRC28 | AMP |
| MEF2A | AMP |
| LYSMD4 | AMP |
| CHSY1 | AMP |
| MYL9 | AMP |
| TGIF2 | AMP |
| C20ORF24 | AMP |
| SLA2 | AMP |
| RHD | AMP |
| PABPN1L | DeepDel |
| TGIF2-C20ORF24 | AMP |
| PDCD1LG2 | AMP |
| FAM169B | AMP |
| PGPEP1L | AMP |
| FRG1B | AMP |
| NDRG3 | AMP |
| DSN1 | AMP |
| CBFA2T3 | DeepDel |
| CD274 | AMP |
| SPATA8 | AMP |
| IGF1R | AMP |
| MIR-3973/3973 | AMP |
| MLLT10P1 | AMP |
| PPAPDC2 | AMP |
| RLN1 | AMP |
| PLGRKT | AMP |
| RIC1 | AMP |
| NR2F2 | AMP |
| MIR1469 | AMP |
| MIR4714 | AMP |
| GLIS3-AS1 | AMP |
| GLIS3 | AMP |
| SLC1A1 | AMP |
| CDC37L1 | AMP |
| AK3 | AMP |
| RCL1 | AMP |
| INSL4 | AMP |
| RLN2 | AMP |
| ERMP1 | AMP |
| KIAA2026 | AMP |
| MLANA | AMP |
| MIR4665 | AMP |
| ARRDC4 | AMP |
| MIR101-2 | AMP |
| MIR-3180-3/5P | AMP |
| SMARCA2 | AMP |
| KCNV2 | AMP |
| KIAA0020 | AMP |
| RFX3 | AMP |
| SPATA6L | AMP |
| JAK2 | AMP |
| RANBP6 | AMP |
| LURAP1L | AMP |
| GLB1L2 | AMP |
| PRMT2 | AMP |
| LINC01235 | AMP |
| VLDLR | AMP |
| INSL6 | AMP |
| KDM4C | AMP |
| TPD52L3 | AMP |
| KMT2A | AMP |
| FOXR1 | AMP |
| LINC00923 | AMP |
| ID4 | AMP |
| MPDZ | AMP |
| LINC00167 | AMP |
| DACH1 | AMP |
| ITPA | AMP |
| SLC4A11 | AMP |
| VLDLR-AS1 | AMP |
| IL33 | AMP |
| UHRF2 | AMP |
| ATP5L | AMP |
| TTC36 | AMP |
| TYRP1 | AMP |
| B3GAT1 | AMP |
| DIP2A | AMP |
| MIR-323/5P | AMP |
| LOC283177 | AMP |
| GLDC | AMP |
| CD3G | AMP |
| UBE4A | AMP |
| ARCN1 | AMP |
| LINC00924 | AMP |
| TMEM45B | AMP |
| NFRKB | AMP |
| PRDM10 | AMP |
| OPCML | AMP |
| SPATA19 | AMP |
| JAM3 | AMP |
| NCAPD3 | AMP |
| VPS26B | AMP |
| THYN1 | AMP |
| ACAD8 | AMP |
| GLB1L3 | AMP |
| SIK1 | AMP |
| COL18A1 | AMP |
| SLC19A1 | AMP |
| S100B | AMP |
| LINC01197 | AMP |
| LOC100128239 | AMP |
| SCN4B | AMP |
| SCN2B | AMP |
| AMICA1 | AMP |
| MPZL3 | AMP |
| MPZL2 | AMP |
| CD3E | AMP |
| CD3D | AMP |
| TMEM25 | AMP |
| IFT46 | AMP |
| PHLDB1 | AMP |
| UPK2 | AMP |
| TMEM261 | AMP |
| PTPRD | AMP |
| LINC00583 | AMP |
| ZDHHC21 | AMP |
| IL10RA | AMP |
| TMPRSS4 | AMP |
| APLP2 | AMP |
| ST14 | AMP |
| ZBTB44 | AMP |
| ADAMTS8 | AMP |
| ADAMTS15 | AMP |
| IGSF9B | AMP |
| COL6A1 | AMP |
| LSS | AMP |
| MCM3AP | AMP |
| C21ORF58 | AMP |
| PCNT | AMP |
| YBEY | AMP |
| CATSPER2 | AMP |
| MCM3AP-AS1 | AMP |
| COL18A1-AS1 | AMP |
| MIR-3680/3680* | AMP |
| TMPRSS4-AS1 | AMP |
| CCDC84 | AMP |
| RPS25 | AMP |
| MIR3656 | AMP |
| TRAPPC4 | AMP |
| ICAM3 | AMP |
| OR1I1 | AMP |
| TPTE | AMP |
| NFIB | AMP |
| CADM1 | AMP |
| MIR3167 | AMP |
| SNX19 | AMP |
| MIR4697 | AMP |
| PCBP3 | AMP |
| COL6A2 | AMP |
| FTCD | AMP |
| SPATC1L | AMP |
| LOC100129027 | AMP |
| MIR4697HG | AMP |
| RPL23AP64 | AMP |
| KIRREL3-AS3 | AMP |
| TREH | AMP |
| CXCR5 | AMP |
| BCL9L | AMP |
| MIR4492 | AMP |
| SLC37A4 | AMP |
| CASP14 | AMP |
| SYDE1 | AMP |
| CER1 | AMP |
| FREM1 | AMP |
| TTC39B | AMP |
| SNAPC3 | AMP |
| LINC00900 | AMP |
| APOA4 | AMP |
| SC5D | AMP |
| HEPACAM | AMP |
| HEPN1 | AMP |
| CCDC15 | AMP |
| SLC37A2 | AMP |
| RPUSD4 | AMP |
| FAM118B | AMP |
| SRPR | AMP |
| FOXRED1 | AMP |
| TIRAP | AMP |
| KIRREL3 | AMP |
| BARX2 | AMP |
| ADARB1 | AMP |
| LINC00315 | AMP |
| POFUT2 | AMP |
| LOC389705 | AMP |
| LOC642852 | AMP |
| DDX6 | AMP |
| ZGLP1 | AMP |
| FDX1L | AMP |
| RAVER1 | AMP |
| TYK2 | AMP |
| CDC37 | AMP |
| MIR1181 | AMP |
| PDE4A | AMP |
| ILVBL | AMP |
| TRAPPC10 | AMP |
| CCDC171 | AMP |
| BUD13 | AMP |
| APOA5 | AMP |
| APOC3 | AMP |
| APOA1 | AMP |
| OAF | AMP |
| ARHGEF12 | AMP |
| TECTA | AMP |
| ROBO3 | AMP |
| ROBO4 | AMP |
| TMEM218 | AMP |
| DCPS | AMP |
| ST3GAL4 | AMP |
| ETS1 | AMP |
| FLI1 | AMP |
| KCNJ1 | AMP |
| KCNJ5 | AMP |
| C11ORF45 | AMP |
| TP53AIP1 | AMP |
| ARHGAP32 | AMP |
| NTM | AMP |
| DSCR8 | AMP |
| PDXK | AMP |
| CSTB | AMP |
| RRP1 | AMP |
| UBE2G2 | AMP |
| SUMO3 | AMP |
| PTTG1IP | AMP |
| ITGB2 | AMP |
| LINC01547 | AMP |
| FAM207A | AMP |
| LINC00163 | AMP |
| LINC00162 | AMP |
| AATBC | AMP |
| ST3GAL4-AS1 | AMP |
| SENCR | AMP |
| WHAMMP1 | AMP |
| ITGB2-AS1 | AMP |
| HYOU1 | AMP |
| VPS11 | AMP |
| HMBS | AMP |
| DPAGT1 | AMP |
| H2AFX | AMP |
| C2CD2L | AMP |
| HINFP | AMP |
| ABCG4 | AMP |
| NLRX1 | AMP |
| PDZD3 | AMP |
| CCDC153 | AMP |
| CBL | AMP |
| ZNF823 | AMP |
| OR7C2 | AMP |
| SLC1A6 | AMP |
| PWP2 | AMP |
| MEF2C | DeepDel |
| PSIP1 | AMP |
| ZPR1 | AMP |
| PAFAH1B2 | AMP |
| SIDT2 | AMP |
| TAGLN | AMP |
| PCSK7 | AMP |
| FXYD2 | AMP |
| FXYD6 | AMP |
| TMPRSS13 | AMP |
| POU2F3 | AMP |
| TMEM136 | AMP |
| NRGN | AMP |
| VSIG2 | AMP |
| ESAM | AMP |
| MSANTD2 | AMP |
| FEZ1 | AMP |
| EI24 | AMP |
| STT3A | AMP |
| CHEK1 | AMP |
| ACRV1 | AMP |
| PATE1 | AMP |
| PATE2 | AMP |
| PATE3 | AMP |
| PATE4 | AMP |
| HYLS1 | AMP |
| PUS3 | AMP |
| DDX25 | AMP |
| CDON | AMP |
| CBR3 | AMP |
| KCNJ15 | AMP |
| DSCR10 | AMP |
| LINC00114 | AMP |
| HMGN1 | AMP |
| RRP1B | AMP |
| C21ORF33 | AMP |
| TRPM2 | AMP |
| LRRC3 | AMP |
| TSPEAR | AMP |
| TSPEAR-AS2 | AMP |
| SSR4P1 | AMP |
| STRC | AMP |
| KRTAP12-2 | AMP |
| KRTAP12-1 | AMP |
| KRTAP10-10 | AMP |
| KRTAP10-4 | AMP |
| KRTAP10-6 | AMP |
| KRTAP10-7 | AMP |
| KRTAP10-9 | AMP |
| KRTAP10-1 | AMP |
| KRTAP10-11 | AMP |
| KRTAP10-2 | AMP |
| KRTAP10-5 | AMP |
| KRTAP10-8 | AMP |
| KRTAP10-3 | AMP |
| KRTAP12-3 | AMP |
| KRTAP12-4 | AMP |
| KRTAP10-12 | AMP |
| LOC100652768 | AMP |
| FXYD6-FXYD2 | AMP |
| LOC649133 | AMP |
| OR8G2 | AMP |
| ACO1 | AMP |
| DDX58 | AMP |
| TOPORS | AMP |
| NDUFB6 | AMP |
| TAF1L | AMP |
| C19ORF66 | AMP |
| ANGPTL6 | AMP |
| PPAN | AMP |
| SNORD105 | AMP |
| P2RY11 | AMP |
| SNORD105B | AMP |
| EIF3G | AMP |
| DNMT1 | AMP |
| S1PR2 | AMP |
| MIR4322 | AMP |
| MRPL4 | AMP |
| ICAM1 | AMP |
| ICAM4 | AMP |
| ICAM5 | AMP |
| KEAP1 | AMP |
| ADGRE5 | AMP |
| DDX39A | AMP |
| CCDC105 | AMP |
| NOTCH3 | AMP |
| AGPAT3 | AMP |
| BNC2 | AMP |
| SIK3 | AMP |
| RNF214 | AMP |
| BACE1 | AMP |
| CEP164 | AMP |
| TRIM29 | AMP |
| GRIK4 | AMP |
| SORL1 | AMP |
| OR8B12 | AMP |
| OR8A1 | AMP |
| SPA17 | AMP |
| DOPEY2 | AMP |
| TTC3 | AMP |
| ERG | AMP |
| PSMG1 | AMP |
| BRWD1 | AMP |
| WRB | AMP |
| B3GALT5-AS1 | AMP |
| ICOSLG | AMP |
| DNMT3L | AMP |
| AIRE | AMP |
| PFKL | AMP |
| C21ORF2 | AMP |
| CKMT1A | AMP |
| TINCR | DeepDel |
| SAFB2 | DeepDel |
| PPAN-P2RY11 | AMP |
| CBR3-AS1 | AMP |
| BRWD1-IT2 | AMP |
| TOPORS-AS1 | AMP |
| BACE1-AS | AMP |
| LTV1 | AMP |
| ZC2HC1B | AMP |
| MCAM | AMP |
| COL5A3 | AMP |
| RDH8 | AMP |
| C3P1 | AMP |
| S1PR5 | AMP |
| DOCK6 | AMP |
| C19ORF80 | AMP |
| ADGRE2 | AMP |
| CNTLN | AMP |
| DSCAML1 | AMP |
| RNF26 | AMP |
| C1QTNF5 | AMP |
| MFRP | AMP |
| USP2 | AMP |
| THY1 | AMP |
| TBCEL | AMP |
| MIR125B1 | AMP |
| BLID | AMP |
| MIRLET7A2 | AMP |
| MIR100 | AMP |
| UBASH3B | AMP |
| CRTAM | AMP |
| C11ORF63 | AMP |
| BSX | AMP |
| HSPA8 | AMP |
| CLMP | AMP |
| MIR4493 | AMP |
| GRAMD1B | AMP |
| SCN3B | AMP |
| PANX3 | AMP |
| TBRG1 | AMP |
| SIAE | AMP |
| PKNOX2 | AMP |
| MORC3 | AMP |
| CHAF1B | AMP |
| CLDN14 | AMP |
| HLCS | AMP |
| RIPPLY3 | AMP |
| PIGP | AMP |
| DSCR9 | AMP |
| DSCR3 | AMP |
| DSCR4 | AMP |
| ETS2 | AMP |
| LCA5L | AMP |
| SH3BGR | AMP |
| SLC37A1 | AMP |
| HYMAI | AMP |
| MIR100HG | AMP |
| USP2-AS1 | AMP |
| MIR-511-2/511 | AMP |
| LOC341056 | AMP |
| SAXO1 | AMP |
| SLC24A2 | AMP |
| KANK2 | AMP |
| ZNF333 | AMP |
| OR7A5 | AMP |
| OR7C1 | AMP |
| OR7A10 | AMP |
| OR7A17 | AMP |
| MIEF1 | DeepDel |
| SH3GL2 | AMP |
| MIR3152 | AMP |
| PLIN2 | AMP |
| PVRL1 | AMP |
| SETD4 | AMP |
| CBR1 | AMP |
| SIM2 | AMP |
| KCNJ6 | AMP |
| TFF1 | AMP |
| TMPRSS3 | AMP |
| UBASH3A | AMP |
| RSPH1 | AMP |
| U2AF1 | AMP |
| CRYAA | AMP |
| LINC00319 | AMP |
| HSF2BP | AMP |
| SYNGR1 | DeepDel |
| TAB1 | DeepDel |
| MGAT3 | DeepDel |
| LOC100133286 | AMP |
| LOC100506472 | DeepDel |
| ADAMTSL1 | AMP |
| ZNF846 | AMP |
| FBXL12 | AMP |
| UBL5 | AMP |
| PIN1 | AMP |
| OLFM2 | AMP |
| ATG4D | AMP |
| MIR1238 | AMP |
| KRI1 | AMP |
| CDKN2D | AMP |
| PKN1 | AMP |
| GIPC1 | AMP |
| PTGER1 | AMP |
| RRAGA | AMP |
| HAUS6 | AMP |
| SCARNA8 | AMP |
| DENND4C | AMP |
| RPS6 | AMP |
| ACER2 | AMP |
| MIR4474 | AMP |
| MIR491 | AMP |
| LINC00840 | DeepDel |
| ZNF202 | AMP |
| OR6X1 | AMP |
| OR6M1 | AMP |
| TMEM225 | AMP |
| OR8D4 | AMP |
| OR4D5 | AMP |
| OR6T1 | AMP |
| OR10S1 | AMP |
| OR10G4 | AMP |
| OR10G9 | AMP |
| OR10G8 | AMP |
| OR10G7 | AMP |
| VWA5A | AMP |
| OR8D1 | AMP |
| OR8D2 | AMP |
| OR8B2 | AMP |
| OR8B3 | AMP |
| OR8B4 | AMP |
| OR8B8 | AMP |
| DYRK1A | AMP |
| B3GALT5 | AMP |
| TFF3 | AMP |
| TFF2 | AMP |
| PDE9A | AMP |
| NDUFV3 | AMP |
| PKNOX1 | AMP |
| CBS | AMP |
| LINC00313 | AMP |
| OR8G1 | AMP |
| OR8G5 | AMP |
| ZNF560 | AMP |
| ZNF426 | AMP |
| ZNF121 | AMP |
| ZNF561 | AMP |
| ZNF561-AS1 | AMP |
| ZNF562 | AMP |
| ZNF812 | AMP |
| AP1M2 | AMP |
| LDLR | AMP |
| SPC24 | AMP |
| ADGRL1 | AMP |
| DNAJB1 | AMP |
| MIR639 | AMP |
| TECR | AMP |
| NDUFB7 | AMP |
| CLEC17A | AMP |
| ADGRE3 | AMP |
| LINC00320 | AMP |
| MLLT3 | AMP |
| MIR4473 | AMP |
| FOCAD | AMP |
| IGSF5 | AMP |
| C2CD2 | AMP |
| ZBTB21 | AMP |
| ABCG1 | AMP |
| WDR4 | AMP |
| ZNF295-AS1 | AMP |
| LOC100507373 | AMP |
| MIR-342/3P | AMP |
| NCAM2 | AMP |
| TUSC1 | AMP |
| MIR802 | AMP |
| TMPRSS2 | AMP |
| LINC00111 | AMP |
| LINC00479 | AMP |
| LINC00112 | AMP |
| PRDM15 | AMP |
| UMODL1 | AMP |
| UMODL1-AS1 | AMP |
| MIR-3911/3911 | AMP |
| CYCS | DeepDel |
| PCP4 | AMP |
| DSCAM | AMP |
| MIR4760 | AMP |
| LINC00323 | AMP |
| BACE2 | AMP |
| MIR3197 | AMP |
| FAM3B | AMP |
| MX2 | AMP |
| MX1 | AMP |
| RIPK4 | AMP |
| CKMT1B | AMP |
| PLAC4 | AMP |
| RUNX1-IT1 | AMP |
| DSCAM-AS1 | AMP |
| LINC00317 | AMP |
| LINC00308 | AMP |
| TMEM50A | AMP |
| OSBPL3 | DeepDel |
| LOC100506422 | AMP |
| SFI1 | DeepDel |
| CAAP1 | AMP |
| IFNAR1 | AMP |
| IFNGR2 | AMP |
| TMEM50B | AMP |
| DNAJC28 | AMP |
| KCNE1 | AMP |
| KCNE2 | AMP |
| SMIM11 | AMP |
| DEPDC5 | DeepDel |
| PISD | DeepDel |
| PRR14L | DeepDel |
| EIF4ENIF1 | DeepDel |
| TIAM1 | AMP |
| SNORA80A | AMP |
| URB1-AS1 | AMP |
| EVA1C | AMP |
| IL10RB | AMP |
| GART | AMP |
| SON | AMP |
| DONSON | AMP |
| CRYZL1 | AMP |
| RCAN1 | AMP |
| CLIC6 | AMP |
| LINC00160 | AMP |
| RUNX1 | AMP |
| D21S2088E | AMP |
| LINC01426 | AMP |
| IL10RB-AS1 | AMP |
| CYYR1 | AMP |
| GRIK1 | AMP |
| SOD1 | AMP |
| SCAF4 | AMP |
| LINC00159 | AMP |
| MIS18A | AMP |
| MRAP | AMP |
| URB1 | AMP |
| PAXBP1 | AMP |
| C21ORF62-AS1 | AMP |
| IFNAR2 | AMP |
| ITSN1 | AMP |
| ATP5O | AMP |
| LINC00649 | AMP |
| MRPS6 | AMP |
| SLC5A3 | AMP |
| LINC00310 | AMP |
| KRTAP8-1 | AMP |
| KRTAP11-1 | AMP |
| KRTAP21-1 | AMP |
| KRTAP21-2 | AMP |
| KRTAP19-8 | AMP |
| GRIK1-AS2 | AMP |
| KRTAP7-1 | AMP |
| ADAMTS1 | AMP |
| ADAMTS5 | AMP |
| MIR4759 | AMP |
| LINC00113 | AMP |
| LINC00314 | AMP |
| LINC00161 | AMP |
| HUNK | AMP |
| C21ORF59 | AMP |
| SYNJ1 | AMP |
| C21ORF62 | AMP |
| OLIG2 | AMP |
| OLIG1 | AMP |
| LINC01548 | AMP |
| KRTAP6-1 | AMP |
| KRTAP6-2 | AMP |
| KRTAP6-3 | AMP |
| KRTAP19-6 | AMP |
| KRTAP19-7 | AMP |
| KRTAP20-1 | AMP |
| KRTAP20-2 | AMP |
| KRTAP22-1 | AMP |
| KRTAP22-2 | AMP |
| KRTAP20-4 | AMP |
| KRTAP20-3 | AMP |
| KRTAP21-3 | AMP |
| PAXBP1-AS1 | AMP |
| MIR155HG | AMP |
| LINC00515 | AMP |
| MRPL39 | AMP |
| JAM2 | AMP |
| ATP5J | AMP |
| GABPA | AMP |
| N6AMT1 | AMP |
| LTN1 | AMP |
| RWDD2B | AMP |
| USP16 | AMP |
| CCT8 | AMP |
| MAP3K7CL | AMP |
| BACH1 | AMP |
| LINC00189 | AMP |
| CLDN17 | AMP |
| LINC00307 | AMP |
| CLDN8 | AMP |
| MIR4327 | AMP |
| TCP10L | AMP |
| KRTAP13-1 | AMP |
| KRTAP15-1 | AMP |
| KRTAP13-4 | AMP |
| KRTAP19-1 | AMP |
| KRTAP13-2 | AMP |
| KRTAP13-3 | AMP |
| KRTAP23-1 | AMP |
| KRTAP19-2 | AMP |
| KRTAP19-3 | AMP |
| KRTAP19-4 | AMP |
| KRTAP19-5 | AMP |
| KRTAP26-1 | AMP |
| KRTAP24-1 | AMP |
| KRTAP27-1 | AMP |
| GRIK1-AS1 | AMP |
| KRTAP25-1 | AMP |
| MIR155 | AMP |
| MIR-372/372 | AMP |
| LINC00158 | AMP |
| APP | AMP |
| LOC339622 | AMP |
